# Mass Spectrometric Analysis of Cucurbitacins and Dihydrocucurbitacins from the Tuber of *Citrullus naudinianus*

**DOI:** 10.3390/biom13081168

**Published:** 2023-07-26

**Authors:** Moritz Benka, Kristof Görlitz, Michael C. Schöttgen, Simon Lagies, Daniel A. Mohl, Michel Kather, Iwanette Du Preez-Bruwer, Davis Mumbengegwi, Robin Teufel, Stefanie Kowarschik, Roman Huber, Dietmar A. Plattner, Bernd Kammerer

**Affiliations:** 1 Core Competence Metabolomics, Hilde-Mangold-Haus, University of Freiburg, 79104 Freiburg, Germany; moritz.benka@ocbc.uni-freiburg.de (M.B.);; 2 Institute of Organic Chemistry, University of Freiburg, 79104 Freiburg, Germany; 3 Hermann Staudinger Graduate School, University of Freiburg, 79104 Freiburg, Germany; 4 Center for Complementary Medicine, Department of Internal Medicine II, University Hospital, Faculty of Medicine, University of Freiburg, 79106 Freiburg, Germany; 5 Institute of Medical Microbiology and Hygiene, Faculty of Medicine, Medical Center—University of Freiburg, 79104 Freiburg, Germany; 6 Centre for Research Services, University of Namibia, Private Bag, Mandume, Ndemufayo Avenue, Pioneers Park, Windhoek 13301, Namibia; 7 Department of Pharmaceutical Sciences, University of Basel, 4056 Basel, Switzerland; 8 BIOSS Centre for Biological Signalling Studies, University of Freiburg, 79104 Freiburg, Germany

**Keywords:** *Citrullus naudinianus*, cucurbitacins, 23,24-dihydrocucurbitacins, HPLC, MS, MS/MS, QTOF, orbitrap

## Abstract

The vast pool of structurally and functionally distinct secondary metabolites (i.e., natural products (NPs)) is constantly being expanded, a process also driven by the rapid progress in the development of analytical techniques. Such NPs often show potent biological activities and are therefore prime candidates for drug development and medical applications. The ethyl acetate extract of the tuber of *Citrullus naudinianus* (*C. naudinianus*), an African melon with edible fruits and seeds, shows in vitro immunomodulatory activity presumably elicited by cucurbitacins that are known major constituents of this plant. Further potentially immunomodulatory cucurbitacins or cucurbitacin derivatives were assumed to be in the tuber. Given the typically high content of cucurbitacins with similar physicochemical features but often distinct bioactivities, an efficient and reliable separation process is a prerequisite for their detailed characterization and assessment in terms of bioactivity. We therefore developed a detection method to screen and differentiate cucurbitacins via high-performance liquid chromatography/quadrupole-time-of-flight tandem mass spectrometry (HPLC-QTOF-MS/MS). In order to confirm the identification, the fragmentation patterns of two cucurbitacins and one 23,24-dihydrocucurbitacin were also investigated. Six characteristic fragments were identified and three of them were employed for the identification of cucurbitacins and 23,24-dihydrocucurbitacins in the extract. As a result, in addition to eight previously reported cucurbitacins from this plant four distinct 23,24-dihydrocucurbitacins (B, D, E, and I) were putatively identified and newly found in the ethyl acetate extract of the tuber of *C. naudinianus*. The established methodology enables rapid and efficient LC-MS-based analysis and identification of cucurbitacins and 23,24-dihydrocucurbitacins in plant extracts.

## 1. Introduction

**Figure 1:**
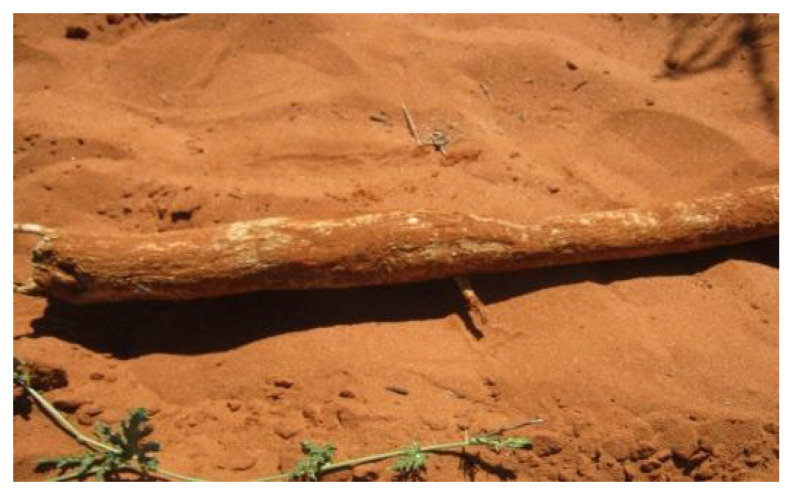
An unearthed tuber of *C. naudinianus*.

*Citrullus naudinianus* (*C. naudinianus*, also known as *Acanthosicyos naudinianus*, *Cucumis naudinianus*, or *Colocynthis naudiniana*) is a member of the *Cucurbitaceae* plant family. The plant is found in Angola, Botswana, Mozambique, Namibia, Zambia, Zimbabwe, and South Africa. Its fruits (Figure 1) are eaten raw or roasted, while the fruit skin and the seeds are typically roasted and pounded before consumption. The tuber is inedible and is used for arrow poisoning by indigenous people [1]. Furthermore, the extract of the tuber shows in vitro immunomodulatory activity suggesting the presence of anti-inflammatory NPs [2].

**Figure 2:**
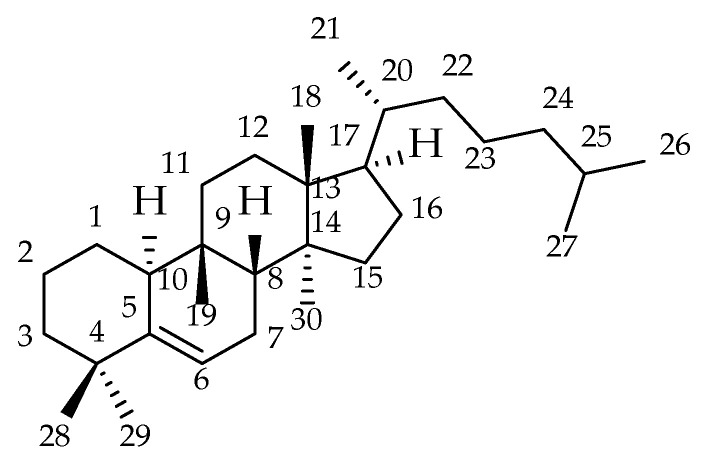
Cucurbitane [19(10 → 9β)abeo-10α-lanost-5-en] skeleton.

Cucurbitacins and their derivatives are responsible for the toxicity and presumably for the tuber’s immunomodulatory effects. It is known to contain the cucurbitacins B, D, E, G, H, I, J, and K in glycosylated as well as non-glycosylated forms, as determined by paper chromatography [3]. Cucurbitacins are bitter-tasting, heavily oxygenated triterpenoid NPs with a tetracyclic steroidal structure derived from the cucurbitane [19(10 → 9β)abeo-10α-lanost-5-en, Figure 2] skeleton and are found in plants of the Cucurbitaceae and others such as the Brassicaceae and the Scrophulariaceae family. The oxygenations may appear at C2, C3, C11, C16, C19, C22, C24, and C25 (Table 1). All of them possess a C-C double bond between C23 and C24 or an O functionality at C24 except cucurbitacin L, cucurbitacin P, and cucurbitacin R, which may be called 23,24-dihydrocucurbitacin I, 23,24-dihydrocucurbitacin O, and 23,24-dihydrocucurbitacin D, respectively. In this paper, the latter labeling for these cucurbitacins as 23,24-dihydrocucurbitacins is applied to show the close relationship to the corresponding unsaturated cucurbitacins. In nature, cucurbitacins and 23,24-dihydrocucurbitacins occur in non-, mono-, or oligo-glycosylated forms [4,5,6].

Several cucurbitacins show pharmacological activities such as antitumor or anti-inflammatory effects. Cucurbitacin B, D, E, and I exhibit considerable inhibitory effects on the growth of human colon (HCT-116), breast (MCF-7), lung (NCI-H460), and central nervous system (SF-268) cancer cell lines. Moreover, all four cucurbitacins exhibit in vitro inhibitory effects on cyclooxygenase-2 (COX-2) at low concentrations while having no effect on cyclooxygenase-1 (COX-1) [7]. Some 23,24-dihydrocucurbitacins show antitumor or anti-inflammatory effects as well, e.g., 23,24-dihydrocucurbitacin B exhibits a notable cytotoxic effect against the cervical (HeLa) cancer cell line and also specifically inhibits COX-2 in mouse macrophages [8,9]. Furthermore, 23,24-dihydrocucurbitacin D (also known as cucurbitacin R) leads to an in vivo reduction of COX-2, nitric oxide synthase-2, tumor necrosis factor-α, and prostaglandin E2, which are all pro-inflammatory [10].

Simple extraction of cucurbitacins from plant material is performed by maceration with sufficiently nonpolar organic solvents such as ethyl acetate [2]. More laborious protocols use a Soxhlet extractor and extract the cucurbitacins with ethyl acetate or ethanol [11,12].

Screening cucurbitacins in biological samples is possible through paper chromatography [3]. The benefit of this method is the simple procedure and its cost efficiency. A large drawback is the need for chemically pure standard compounds to compare the retention factors and the imprecise separation of the analytes.

A newer approach for screening is the separation of the analytes through preparative column chromatography and the isolation of the fractions followed by a nuclear magnetic resonance (NMR) analysis. There is potentially no limit for the sample amount using preparative columns. However, often the separation is not precise enough, so the isolated fractions of the chromatography have to be separated again with another column or solvent. In case the sample is pure and its molecular formula (identified by another method) is known, the chemical structure of the analytes is accessible through NMR. With this method, 23,24-dihydrocucurbitacins can also be identified in contrast to the screening through paper chromatography, which lacks a good separation of the cucurbitacin and its 23,24-dihydro form [12]. The drawback of this method is the need for comparatively huge amounts of the (pure) analyte and the lengthy separation process.

The perhaps most elegant screening method because of the small sample size and the absence of the need for the isolation of fractions is high-performance liquid chromatography (HPLC) coupled with mass spectrometry (MS) and tandem mass spectrometry (MS/MS), respectively. A gradient of a polar mobile phase containing, e.g., water, ammonium formate, and formic acid, and a sufficiently nonpolar mobile phase containing, e.g., acetonitrile, ammonium formate, and formic acid, leads to a separation of cucurbitacins [13]. With the chemically pure standards, the retention times during chromatography and the fragmentation patterns are analyzed. With these insights, it should be possible to identify and differentiate between cucurbitacins and 23,24-dihydrocucurbitacins, which primarily differ in the number and position of their C-C double bonds.

Due to the highly promising pharmacological activities of cucurbitacins and 23,24-dihydrocucurbitacins, the identification and analysis of plants containing these NPs is a worthwhile task, especially considering the total lack of reported total syntheses for cucurbitacins so far. A prime example and promising candidate is the tuber of *C. naudinianus*, which is known to contain cucurbitacins. Since existing information is solely based on paper chromatography [3], the aim of this study was to establish an HPLC-MS/MS-based workflow to reliably differentiate and separate cucurbitacins and 23,24-dihydrocucurbitacins.

## 2. Materials and Methods

### 2.1. Chemicals

Ethyl acetate in HPLC isocratic grade was purchased from Bernd Kraft GmbH. Acetonitrile, isopropanol, and formic acid in LC-MS grade were purchased from VWR International GmbH (Bruchsal, Germany). Deionized water was purified using a PureLab Flex system. Cucurbitacin B and E were purchased from abcr GmbH (Karlsruhe, Germany). Hemslecin A (25-O-acetyl-23,24-dihydrocucurbitacin F) was purchased from BLD Pharmatech GmbH (Kaiserslautern, Germany).

### 2.2. Plant Material, Extraction and Sample Preparation

The tuber of *C. naudinianus* was collected in the Ohangwena region in Namibia. The dried tuber (3.0 g) was ground and extracted with ethyl acetate (30 mL) at room temperature for 24 h while being stirred by a magnetic stirrer. The resulting suspension was filtered. The filter cake was extracted again twice as described. The solvent of the filtrates was removed under vacuum at 40 °C to obtain the dry extract. The extraction protocol is based on the protocol used in [2].

The dry extract (10 mg) was dissolved in isopropanol (1.0 mL) to obtain a stock solution. The stock solution (1.0 µL) was mixed with a solution of water and acetonitrile (99 µL, 98:2) and centrifuged (10 min, 20,000× *g*, 4 °C). The supernatant (90 µL) was withdrawn to receive the ready sample. The residue was discarded. The same procedure was performed to obtain a sample of each standard compound.

### 2.3. Flow Injection and HPLC Conditions

The flow injection analysis of the standards was performed using a 1200 HPLC system (Agilent Technologies, Waldbronn, Germany) without a column using a solvent mixture of water + 0.1% FA (A) and acetonitrile:isopropanol (9:1) + 0.1% FA (B) at a ratio of A:B = 1:1. The flow rate was set to 0.6 mL/min. The sample injection volume was set to 5 µL.

The HPLC analysis was performed using the same system equipped with the ZORBAX Eclipse Plus C18 RRHD Column (50 × 3 mm; 1.8 µm, Agilent Technologies, Waldbronn, Germany) using the same solvents A and B. The following gradient was applied: 0–1 min, 2% B; 1–3 min, 20% B; 3–13 min, 35% B; 13–19 min, 65% B; 19–22 min, 98% B; 22–29 min, 98% B; 29–30 min, 2% B; 30–35 min, 2% B. The column temperature was maintained at 40 °C, and the flow rate was set to 0.6 mL/min. The sample injection volume was set to 5 µL.

### 2.4. ESI-QTOF-MS/MS and ESI-Orbitrap-MS conditions

MS and MS/MS experiments were performed using a 6520 accurate-Mass Q-TOF-MS system (Agilent Technologies, Waldbronn, Germany) in negative electrospray ionization mode with a mass resolution of 20,000. The full-scan data were collected between *m/z* 100 and 1200 in centroid mode. The following MS parameters were applied: Gas temperature, 325 °C; drying gas flow, 8 L/min; nebulizer pressure, 40 psig; capillary voltage, 3000 V; fragmentor voltage, 135 V; skimmer voltage, 65 V; OCT 1 RF Vpp, 750 V. The mass spectrometer was calibrated internally using reference masses at *m/z* 112.9856 ([C_2_HF_3_O_2_ − H]^−^) and 1033.9881 ([C_18_H_18_F_24_N_3_O_6_P_3_ + C_2_HF_3_O_2_ − H]^−^) to obtain high-accuracy mass measurements. The MS/MS experiments were operated using 40 eV as the collision energy and a mass window of *m/z* 1.3. Nitrogen was used as the collision gas.

Further MS experiments were performed using an LTQ Orbitrap XL (Thermo Fisher Scientific, Bremen, Germany) in negative electrospray ionization mode with a mass resolution of 60,000. The full-scan data were collected between *m/z* 100 and 1200 in centroid mode. The following MS parameters were applied: Capillary temperature, 300 °C; sheath gas flow, 32 L/min; aux gas flow, 10 L/min, sweep gas flow, 5 L/min; source voltage, 4.5 kV; source current, 100 µA; capillary voltage, −48 V; tube lens, 112.64 V. The Orbitrap was calibrated internally using polyfluorinated hydrocarbons as reference masses at *m/z* 130.9926 ([C_3_HF_5_ − H]^−^), 180.9894 ([C_4_HF_7_ − H]^−^, 230.9862 ([C_5_HF_9_ − H]^−^), 280.983 ([C_6_HF_11_ − H]^−^), 330.9798 ([C_7_HF_13_ − H]^−^), 380.9766 ([C_8_HF_15_ − H]^−^), 430.9734 ([C_9_HF_17_ − H]^−^), 480.9702 ([C_10_HF_19_ − H]^−^), 530.9670 ([C_11_HF_21_ − H]^−^), 580.9638 ([C_12_HF_23_ − H]^−^), 630.9606 ([C_13_HF_25_ − H]^−^), 680.9574 ([C_14_HF_27_ − H]^−^), 730.9542 ([C_15_HF_29_ − H]^−^), 780.9510 ([C_16_HF_31_ − H]^−^), and 830.9479 ([C_17_HF_33_ − H]^−^) to obtain high-accuracy mass measurements.

### 2.5. Data Analysis

The analysis of the flow injection-QTOF-MS/MS and HPLC-QTOF-MS/MS data was performed using the Qualitative Analysis B.07.00 software from Agilent. Compounds were found by molecular features. The data range was restricted to retention times between 3 and 28 min and to *m/z* between 100 and 1200 with a minimum signal height of 10,000 counts to qualify as a compound. The formulas were generated using only C, H, and O atoms in anticipation of cucurbitacins and their glycosides. The signal spacing tolerance was set to 5 ppm.

The analysis of the HPLC-Orbitrap-MS data was performed using the Thermo Xcali-bur 3.0.63.3 software from Thermo Fisher Scientific. Compounds were found manually.

## 3. Results

### 3.1. Extraction of Natural Products

Three tuber samples (each 3.00 g) were extracted as described in Chapter 2.2. In each case, a yellow amorphous solid (191, 191, and 182 mg) was obtained, which corresponds to approximately 6% of each initial tuber mass.

### 3.2. QTOF-MS/MS Behavior of Flow-Injected Cucurbitacin and 23,24-Dihydrocucurbitacin Standards

The negative ionization mode was chosen to analyze cucurbitacins and 23,24-dihydrocucurbitacins because of the better signal-to-noise ratio than in the positive mode [13]. [M − H]^−^, [M + Cl]^−^, [M + FA − H]^−^, and [M + NO_3_]^−^ adducts were detected (Figures S1–S3 in the Supplementary Materials). Additionally, for each standard, a signal was found, which could be related to [M − C_2_H_4_O_2_ − H]^−^ and presumably corresponds to the in-source fragment of the standard after the elimination of acetic acid at the tail moiety (vide infra).

**Figure 3:**
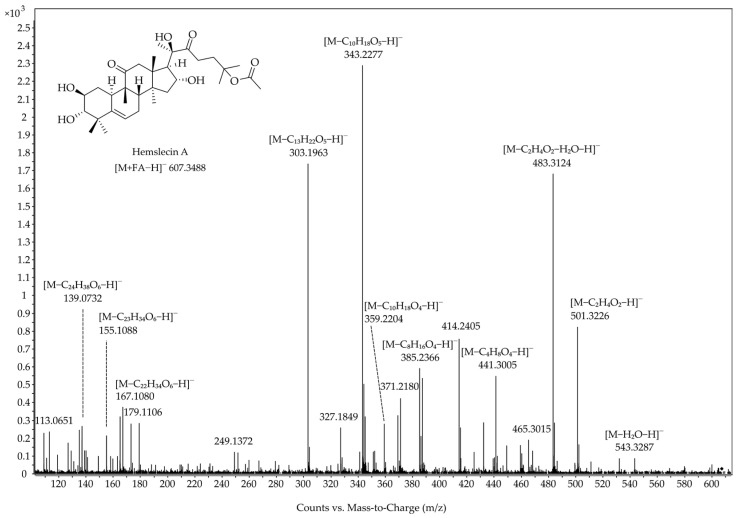
MS/MS spectrum of the hemslecin A standard [M + FA − H]^−^ adduct (flow injection, negative mode, 40 eV collision energy).

The fragmentation of the [M + FA − H]^−^ adduct of hemslecin A at a collision energy of 40 eV led to characteristic signals (Figure 3) that could be related to [M − H_2_O − H]^−^, [M − C_2_H_4_O_2_ − H]^−^, [M − C_2_H_4_O_2_ − H_2_O − H]^−^, [M − C_4_H_8_O_4_ − H]^−^, [M − C_8_H_16_O_4_ − H]^−^, [M − C_10_H_18_O_4_ − H]^−^, [M − C_10_H_18_O_5_ − H]^−^, [M − C_13_H_22_O_5_ − H]^−^, [M − C_22_H_34_O_6_ − H]^−^, [M − C_23_H_34_O_6_ − H]^−^, and [M − C_24_H_38_O_6_ − H]^−^.

**Figure 4:**
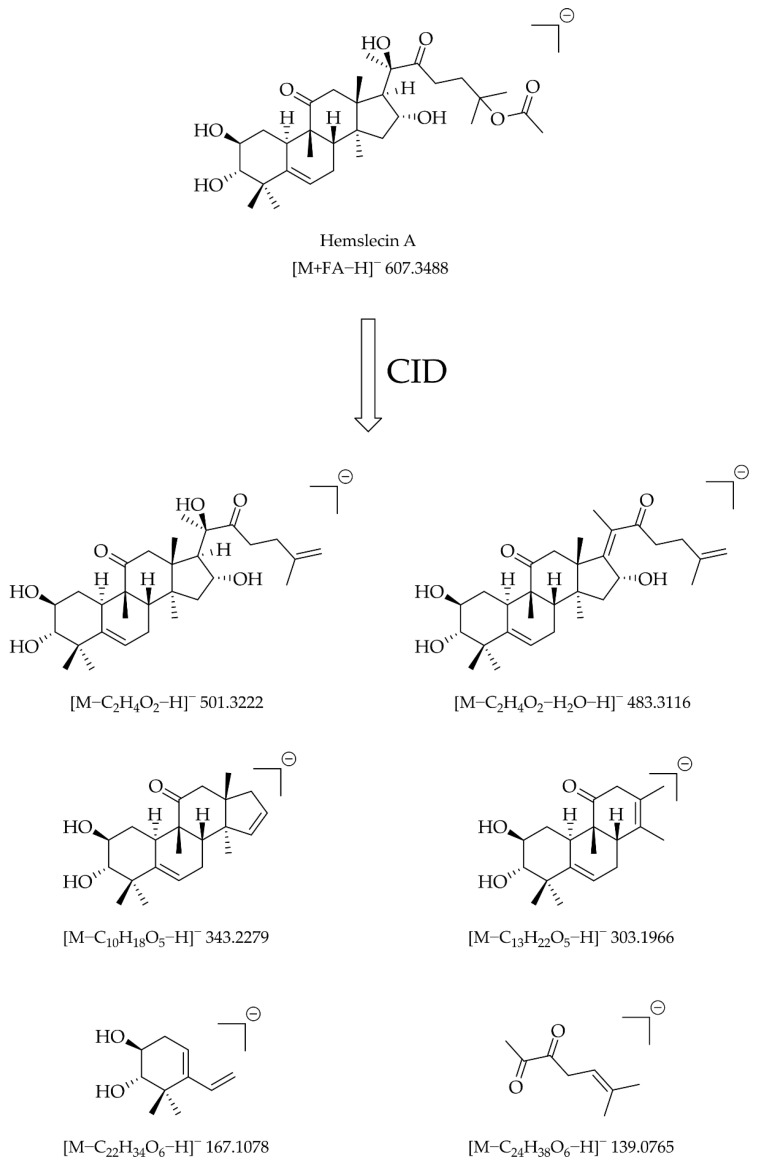
Putative fragment structures with their theoretical *m/z* value resulting from CID (40 eV) of hemslecin A.

Comparing these signals to the signals of cucurbitacin B and E (Figures S4 and S5 in the Supplementary Materials), we could assign signals to defined fragments (Figure 4). The easiest fragment signal to explain is [M − C_2_H_4_O_2_ − H]^−^ since the loss corresponds to the elimination of acetic acid at the tail moiety. It was assumed that the elimination of water in the fragment corresponding to the [M − C_2_H_4_O_2_ − H_2_O − H]^−^ signal occurred at C17/C20, as it gives rise to an energetically favorable conjugated system of C-C double bonds. The loss in the [M − C_10_H_18_O_5_ − H]^−^ fragment signal corresponds to an elimination of the ten C atoms (plus the bound H and O atoms) of the tail moiety in addition to the elimination of water likely at C15/C16. The [M − C_13_H_22_O_5_ − H]^−^ signal presumably corresponds to the cucurbitacin with a cleaved ring D and the [M − C_22_H_34_O_6_ − H]^−^ signal to the compound with a cleaved ring B via a retro Diels–Alder reaction. The [M − C_24_H_38_O_6_ − H]^−^ signal corresponds to the tail moiety and thus it is the counterpart of the [M − C_10_H_18_O_5_ − H]^−^ fragment signal.

The [M − C_2_H_4_O_2_ − H]^−^, the [M − C_13_H_22_O_5_ − H]^−^, and the [M − C_22_H_34_O_6_ − H]^−^ signals allow a reasonably solid assignment of the C-C double bond positions and are the basis for the screening of cucurbitacins and 23,24-dihydrocucurbitacins in *C. naudinianus*.

### 3.3. HPLC-QTOF-MS Analysis of the Extract, HPLC-QTOF-MS/MS Screening of Cucurbitacins and 23,24-Dihydrocucurbitacins, and HPLC-Orbitrap-MS Analysis of the Extract

**Figure 5:**
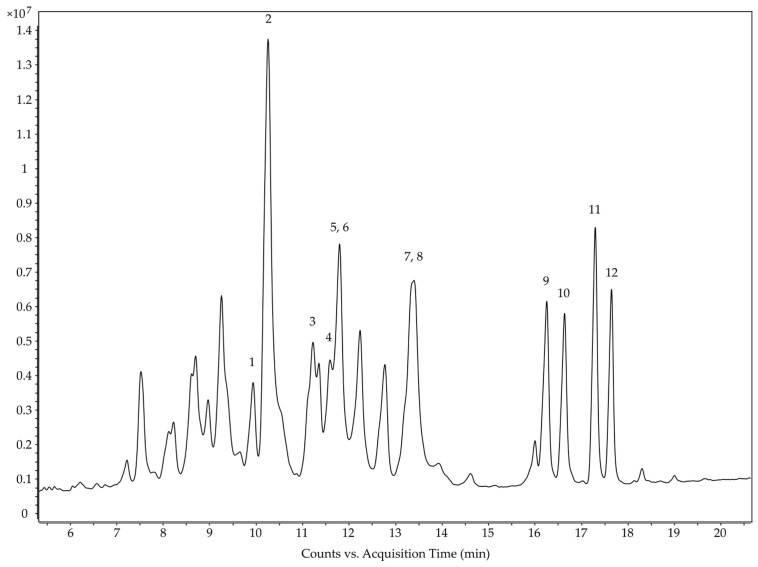
Section of the total ion current (TIC) chromatogram of the HPLC-QTOF-MS analysis of the ethyl acetate extract of the tuber of *C. naudinianus* with numbered peaks (see Figure 6 and Table 2). HPLC settings: ZORBAX Eclipse Plus C18 RRHD Column (50 × 3 mm; 1.8 µm); solvent gradient of water + 0.1% FA (A) and acetonitrile:isopropanol (9:1) + 0.1% FA (B); column temperature, 40 °C; flow rate 0.6 mL/min. MS settings: Negative mode, capillary voltage, 3000 V; fragmentor voltage, 135 V; skimmer voltage, 65 V.

The HPLC-MS protocol was developed and optimized using an Eclipse Plus C18 RRHD Column at 40 °C. A solvent gradient of water + 0.1% FA (A) and acetonitrile:isopropanol (9:1) + 0.1% FA (B) was applied with a stable flow rate of 0.6 mL/min. A relatively high percentage of B in an early stage of the chromatography (starting at 2% for one minute to 20% two minutes later) followed by the first flat but progressively rising solvent gradient resulted in an early elution and adequate separation of the most interesting analytes. The resulting total ion current (TIC) chromatogram is shown in Figure 5 (see Figure S6 in the Supplementary Materials for the full TIC chromatogram of the extract).

A total of 95 compounds were found to which a [C,H,O] molecular formula was assigned. In many cases, the software did not identify the ion as the [M − H]^−^, the [M + Cl]^−^ adduct, or the [M + FA − H]^−^ adduct ion. Therefore, the spectrograms of all features were reviewed and manually searched for the signals of the [M − H]^−^, [M + Cl]^−^, and [M + FA − H]^−^ adducts. For this purpose, the [M + ^35^Cl]^−^, [M + ^37^Cl]^−^, and [M + FA − H]^−^ adduct signals proved especially helpful, allowing the curation of the results in this manner (Table S1 in the Supplementary Materials).

Most compounds contain 36, 38, 42, or 44 C atoms. Since the MS/MS spectra of these compounds show signals corresponding to the loss of a hexose ([M − C_6_H_10_O_5_ − H]^−^) or of a deoxyhexose ([M − C_6_H_10_O_4_ − H]^−^) moiety and often a mass loss as described in chapter 3.2, it can be inferred that these compounds represent glycosides of cucurbitacins and 23,24-dihydrocucurbitacins. It was not possible to identify the structure of the (deoxy)hexose moieties and their positions in the analytes. In addition to these mass losses, some compounds produced fragments that indicated a loss of C_6_H_12_O_3_.

**Figure 6:**
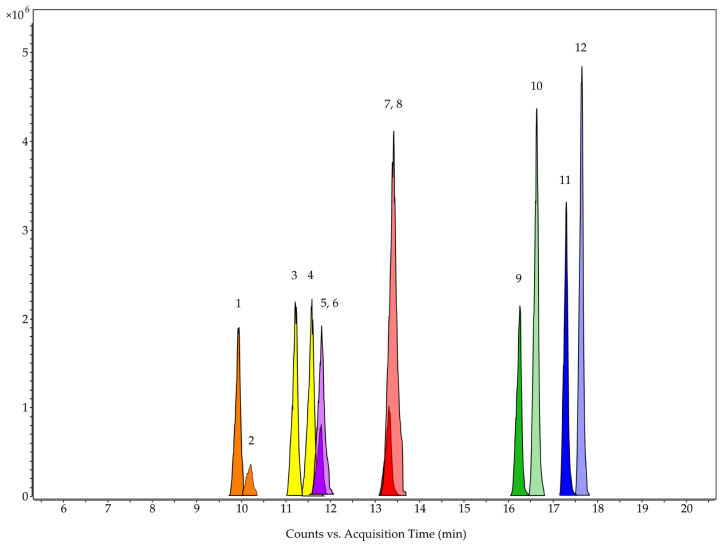
Extracted compound chromatograms (ECCs) of cucurbitacin G/H (orange, 1 and 2), J/K (yellow, 3 and 4), D (purple, 5), I (red, 7), B (green, 9), and E (blue, 11) and of 23,24-dihydrocucurbitacin D (light purple, 6), I (light red, 8), B (light green, 10), and E (light blue, 12) of the HPLC-QTOF-MS analysis of the ethyl acetate extract of the tuber of *C. naudinianus* (see Table 2). HPLC settings: ZORBAX Eclipse Plus C18 RRHD Column (50 × 3 mm; 1.8 µm); solvent gradient of water + 0.1% FA (A) and acetonitrile:isopropanol (9:1) + 0.1% FA (B); column temperature, 40 °C; flow rate 0.6 mL/min. MS settings: Negative mode; capillary voltage, 3000 V; fragmentor voltage, 135 V; skimmer voltage, 65 V.

**Figure 7:**
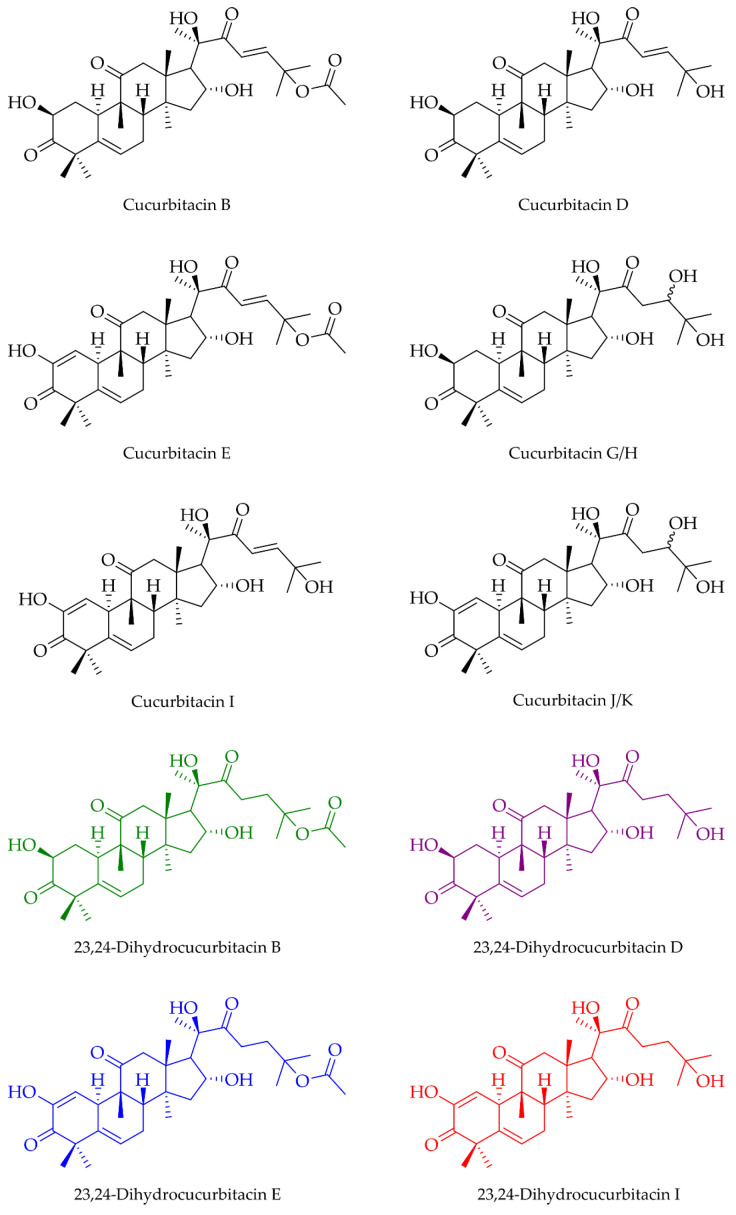
Known cucurbitacins and newly found 23,24-dihydrocucurbitacins B (green), D (purple), E (blue), and I (red) putatively identified in the tuber of *C. naudinianus*.

The majority of the remaining compounds contain 30 or 32 C atoms. Many of them showed mass losses corresponding to the three fragments that are sufficient for screening cucurbitacins and 23,24-dihydrocucurbitacins described in chapter 3.2 (Figures S7–S18 in the Supplementary Materials). In this manner, a total of twelve cucurbitacins and 23,24-dihydrocucurbitacins were putatively identified, whereby it was not possible to distinguish cucurbitacin G and H, as well as cucurbitacin J and K (Figure 6 and Table 2). In addition to the known cucurbitacins B, D, E, G, H, I, J, and K, 23,24-dihydrocucurbitacin B, D, E, and I were found (Figure 7). Thus, every cucurbitacin with a Δ^23^ C-C double bond also occurred in its 23,24-dihydro form.

**Figure 8:**
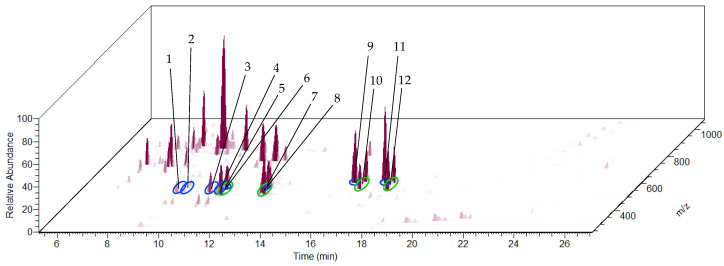
Section of the total ion current (TIC) chromatogram of the HPLC-Orbitrap-MS analysis of the ethyl acetate extract of the tuber of *C. naudinianus* with numbered peaks of the found cucurbitacins (encircled in blue) or 23,24-dihydrocucurbitacins (encircled in green). HPLC settings: ZORBAX Eclipse Plus C18 RRHD Column (50 × 3 mm; 1.8 µm); solvent gradient of water + 0.1% FA (A) and acetonitrile:isopropanol (9:1) + 0.1% FA (B); column temperature, 40 °C; flow rate 0.6 mL/min. Orbitrap-MS settings: Negative mode, source voltage, 4.5 kV; source current, 100 µA; capillary voltage, −48 V.

The HPLC-MS analysis was conducted again using LTQ Orbitrap XL to confirm the QTOF-MS data regarding the mass accuracy and the generation of the molecular formulas (Figure 8). The signals of the [M + FA − H]^−^ adducts of the cucurbitacins and 23,24-dihydrocucurbitacins were searched and found (Table 3). The Orbitrap data showed a higher mass accuracy. The comparison to the QTOF-MS data confirmed the generated molecular formulas.

## 4. Discussion

### 4.1. Extraction of Natural Products

The extraction of the tuber with ethyl acetate leads to an extract containing the desired cucurbitacins and the putatively identified 23,24-dihydrocucurbitacins. In addition, compounds were extracted, seeming to be glycosides of cucurbitacins and 23,24-dihydrocucurbitacins. There are no data to compare the mass of the extract of the tuber of *C. naudinianus* using ethyl acetate. Considering the extracted mass of 6% of the starting material, it seems to be an adequate extraction method. Ethanol as the extraction solvent was expected to yield an extract with a higher mass caused by a much higher content of less interesting polar compounds [12].

### 4.2. QTOF-MS/MS Behavior of Flow-Injected Cucurbitacin and 23,24-Dihydrocucurbitacin Standards

The most important structural differences between the several cucurbitacins are the number and position of their C-C double bonds. Since *C. naudinianus* is known to contain cucurbitacin B and E (in addition to cucurbitacin D, G, H, I, J, and K), which can be purchased and differ in the presence of the Δ^1^ C-C double bond, these two were chosen as fragmentation standards. To confirm the fragmentation hypotheses and to analyze a 23,24-dihydrocucurbitacin, hemslecin A (25-O-acetyl-23,24-dihydrocucurbitacin F) was fragmented as well.

MS analyses of the standard compounds showed [M − H]^−^, [M + FA − H]^−^, and [M − C_2_H_4_O_2_ − H]^−^ adducts, which were expected [13]. In addition, [M + Cl]^−^ and [M + NO_3_]^−^ adducts were detected. The presence of Cl^−^ and NO_3_^−^ ions was attributed to sample contamination. The reason for the noticeably high [M + NO_3_]^−^ adduct signal in the hemslecin A sample (Figure S3) is not clear. It may be caused by a higher NO_3_^−^ concentration or by a stronger ionization of that adduct compared to the other adducts.

For the MS/MS experiments, the [M + FA − H]^−^ adducts were chosen as the standard fragmentation adducts, as they exhibited superior signal heights and yielded interpretable fragmentation patterns unlike the [M + Cl]^−^ or the [M + NO_3_]^−^ adducts. The superior signal heights of the [M + FA − H]^−^ adducts are typical for cucurbitacins [13]. Low collision (<40 eV) energies lead to only a few signals. High collision energies (>40 eV) lead to many signals but many of them are not interpretable anymore. We found that a collision energy of 40 eV leads to decent fragmentation with many interpretable signals. It was possible to assign some of these signals to defined fragments considering classical chemical mechanisms such as eliminations and the retro Diels–Alder reaction. More fragment signals were found, but most of them seemed to be caused by rearrangement reactions, which is much less easy to explain so we were content with the easily assigned fragment signals. These were a satisfactory base for the further screening of cucurbitacins and 23,24-dihydrocucurbitacins.

### 4.3. HPLC-QTOF-MS Analysis of the Extract, HPLC-QTOF-MS/MS Screening of Cucurbitacins and 23,24-Dihydrocucurbitacins, and HPLC-Orbitrap-MS Analysis of the Extract

The development and optimization of the HPLC method started with a simple linear solvent gradient of water + 0.1% FA and acetonitrile + 0.1% FA. A high stable flow rate of 0.6 mL/min was set to maintain high pressures, thereby resulting in desirably narrow peaks. Flattening the solvent gradient in the first half and a steepening in the second half of the chromatography and the addition of isopropanol to the acetonitrile + 0.1% FA solvent led to a better separation of the analytes, especially of cucurbitacin B and E and their 23,24-dihydro forms in the same time. A flatter gradient allows the analytes with almost the same polarity to separate. The isopropanol modulates the polarity of the solvent and thus leads to an even more appropriate separation of the analytes. 

Under the assumption that only cucurbitacins and their glycosides were present, a total of 95 compounds were found to which a [C,H,O] molecular formula could be assigned. The data suggested many glycosides in the extract. However, we decided to disregard them because it was not possible to identify the position of the sugar moiety in the analyte by HPLC-QTOF-MS/MS. Most of the remaining compounds contained 30 or 32 C atoms, which is typical and showed the typical fragmentation behavior of cucurbitacins and 23,24-dihydrocucurbitacins.

## 5. Conclusions

A high-performance liquid chromatography (HPLC) method combined with a quadrupole-time-of-flight/tandem mass spectrometry (QTOF-MS/MS) method was developed to analyze the ethyl acetate extract of the tuber of *C. naudinianus*. This method was confirmed using HPLC-Orbitrap-MS. Characteristic fragmentation patterns of two cucurbitacins and one 23,24-dihydrocucurbitacin standard were first verified, which allowed the putative determination of hallmark fragments that could be used for the previous identification of unknown 23,24-dihydrocucurbitacins in the tuber of *C. naudinianus*. Taken together, QTOF-MS/MS coupled with HPLC enabled the separation and identification of the four newly found 23,24-dihydrocucurbitacins B, D, E, and I in the ethyl acetate extract of the tuber of *C. naudinianus* in addition to the eight known cucurbitacins B, D, E, G, H, I, J, and K. These results form the basis for the future assessment of these cucurbitacins and 23,24-dihydrocucurbitacins with respect to their bioactivities and potential as drug leads.

## Figures and Tables

**Table 1:** Functional groups of cucurbitacins.

Cucurbitacin	Position and Type of O-Functionalities in the Cucurbitane Skeleton									Position of C-C Double Bonds in the Cucurbitane Skeleton		
	2	3	11	16	19	20	22	24	25	1	5	23
A	OH_β_	=O	=O	OH_α_	OH	OH	=O		OAc		Δ^5^	Δ^23^
B	OH_β_	=O	=O	OH_α_		OH	=O		OAc		Δ^5^	Δ^23^
C		OH_α_	=O	OH_α_	OH	OH	=O		OAc		Δ^5^	Δ^23^
D	OH_β_	=O	=O	OH_α_		OH	=O		OH		Δ^5^	Δ^23^
E	OH	=O	=O	OH_α_		OH	=O		OAc	Δ^1^	Δ^5^	Δ^23^
F	OH_β_	OH_α_	=O	OH_α_		OH	=O		OH		Δ^5^	Δ^23^
G	OH_β_	=O	=O	OH_α_		OH	=O	(24*R*)-OH	OH		Δ^5^	
H	OH_β_	=O	=O	OH_α_		OH	=O	(24*S*)-OH	OH		Δ^5^	
I	OH	=O	=O	OH_α_		OH	=O		OH	Δ^1^	Δ^5^	Δ^23^
J	OH	=O	=O	OH_α_		OH	=O	(24*R*)-OH	OH	Δ^1^	Δ^5^	
K	OH	=O	=O	OH_α_		OH	=O	(24*S*)-OH	OH	Δ^1^	Δ^5^	
L	OH	=O	=O	OH_α_		OH	=O		OH	Δ^1^	Δ^5^	
O	OH_α_	OH_α_	=O	OH_α_		OH	=O		OH		Δ^5^	Δ^23^
P	OH_α_	OH_α_	=O	OH_α_		OH	=O		OH		Δ^5^	
Q	OH_α_	OH_α_	=O	OH_α_		OH	=O		OAc		Δ^5^	Δ^23^
R	OH_β_	=O	=O	OH_α_		OH	=O		OH		Δ^5^	
S	OH	=O	=O	O_α_-C24			=O	(24*S*)-O-C16	OH	Δ^1^	Δ^5^	
T	OH	=O	=O	O_α_-C24		OH	=O	(24*S*)-O-C16	OMe	Δ^1^	Δ^5^	

**Table 2:** Known cucurbitacins and newly found 23,24-dihydrocucurbitacins putatively identified in the tuber of *C. naudinianus* using HPLC-QTOF-MS/MS.

Peak No. in Figure 5/Figure 6	Name	Molecular Formula	RT (min)	*m/z*	Ion Type	Exact *m/z*	Error (ppm)	MS/MS *m/z* of [M + FA − H]^−^
1	Cucurbitacin G/H	C_30_H_46_O_8_	9.93	579.3178	[M + FA − H]^−^	579.3175	0.52	497.2708;
				569.2898	[M + Cl]^−^	569.2887	1.93	301.1792;
				533.3130	[M − H]^−^	533.3120	1.88	165.0914.
2	Cucurbitacin G/H	C_30_H_46_O_8_	10.19	579.3179	[M + FA − H]^−^	579.3175	0.69	497.2931;
				569.2895	[M + Cl]^−^	569.2887	1.41	301.1826;
				533.3127	[M − H]^−^	533.3120	1.31	165.0934.
3	Cucurbitacin J/K	C_30_H_44_O_8_	11.21	577.3029	[M + FA − H]^−^	577.3018	1.91	495.2697;
				567.2749	[M + Cl]^−^	567.2730	3.35	299.1601;
				531.2980	[M − H]^−^	531.2963	3.20	163.0751.
4	Cucurbitacin J/K	C_30_H_44_O_8_	11.57	577.3030	[M + FA − H]^−^	577.3018	2.08	495.2789;
				567.2746	[M + Cl]^−^	567.2730	2.82	299.1673;
				531.2974	[M − H]^−^	531.2963	2.07	163.0775.
5	Cucurbitacin D	C_30_H_44_O_7_	11.77	561.3090	[M + FA − H]^−^	561.3069	3.74	497.2872;
				551.2796	[M + Cl]^−^	551.2781	2.72	301.1795;
				515.3014	[M-H]^−^	515.3003	2.13	165.0909.
6	23,24-Dihydro-cucurbitacin D	C_30_H_46_O_7_	11.80	563.3243	[M + FA − H]^−^	563.3226	3.02	499.3082;
				553.2935	[M + Cl]^−^	553.2938	−0.54	4301.1792;
				517.3187	[M − H]^−^	517.3171	3.09	165.0918.
7	Cucurbitacin I	C_30_H_42_O_7_	13.31	559.2930	[M + FA − H]^−^	559.2913	3.04	495.2708;
				549.2641	[M + Cl]^−^	549.2625	2.91	299.1670;
				513.2864	[M − H]^−^	513.2858	1.17	163.0743.
8	23,24-Dihydro-cucurbitacin I	C_30_H_44_O_7_	13.41	561.3085	[M + FA − H]^−^	561.3069	2.85	497.2943;
				551.2783	[M + Cl]^−^	551.2781	0.36	299.1745;
				515.3030	[M − H]^−^	515.3014	3.10	163.0764.
9	Cucurbitacin B	C_32_H_46_O_8_	16.24	603.3190	[M + FA − H]^−^	603.3175	2.49	497.2901;
				593.2902	[M + Cl]^−^	593.2887	2.53	301.1423;
				557.3102	[M − H]^−^	557.3120	−3.23	165.0911.
10	23,24-Dihydro-cucurbitacin B	C_32_H_48_O_8_	16.63	605.3345	[M + FA − H]^−^	605.3331	2.31	499.3121;
				595.3055	[M + Cl]^−^	595.3043	2.02	301.1763;
				559.3282	[M − H]^−^	559.3276	1.07	165.0914.
11	Cucurbitacin E	C_32_H_44_O_8_	17.29	601.3034	[M + FA − H]^−^	601.3018	2.66	495.2737;
				591.2745	[M + Cl]^−^	591.2730	2.54	299.1276;
				555.2960	[M − H]^−^	555.2963	−0.54	163.0751.
12	23,24-Dihydro-cucurbitacin E	C_32_H_46_O_8_	17.64	603.3185	[M + FA − H]^−^	603.3175	1.66	497.2902;
				593.2895	[M + Cl]^−^	593.2887	1.35	299.1663;
				557.3126	[M − H]^−^	557.3120	1.08	163.0771.

**Table 3:** Known cucurbitacins and newly found 23,24-dihydrocucurbitacins putatively identified in the tuber of *C. naudinianus* using HPLC-Orbitrap-MS.

Peak No. in Figure 8	Name	Molecular Formula	RT (min)	*m/z*	Ion Type	Exact *m/z*	Error (ppm)
1	Cucurbitacin G/H	C_30_H_46_O_8_	9.18	579.3170	[M + FA − H]^−^	579.3175	−0.86
2	Cucurbitacin G/H	C_30_H_46_O_8_	9.48	579.3172	[M + FA − H]^−^	579.3175	−0.52
3	Cucurbitacin J/K	C_30_H_44_O_8_	10.50	577.3014	[M + FA − H]^−^	577.3018	−0.69
4	Cucurbitacin J/K	C_30_H_44_O_8_	10.88	577.3013	[M + FA − H]^−^	577.3018	−0.87
5	Cucurbitacin D	C_30_H_44_O_7_	11.01	561.3066	[M + FA − H]^−^	561.3069	−0.53
6	23,24-Dihydrocucurbitacin D	C_30_H_46_O_7_	11.01	563.3220	[M + FA − H]^−^	563.3226	−1.07
7	Cucurbitacin I	C_30_H_42_O_7_	12.63	559.2911	[M + FA − H]^−^	559.2913	−0.36
8	23,24-Dihydrocucurbitacin I	C_30_H_44_O_7_	12.71	561.3064	[M + FA − H]^−^	561.3069	−0.89
9	Cucurbitacin B	C_32_H_46_O_8_	15.83	603.3172	[M + FA − H]^−^	603.3175	−0.50
10	23,24-Dihydrocucurbitacin B	C_32_H_48_O_8_	16.26	605.3329	[M + FA − H]^−^	605.3331	−0.33
11	Cucurbitacin E	C_32_H_44_O_8_	17.03	601.3018	[M + FA − H]^−^	601.3018	0.00
12	23,24-Dihydrocucurbitacin E	C_32_H_46_O_8_	17.36	603.3175	[M + FA − H]^−^	603.3175	0.00

## Data Availability

The data are shown in the article and in the Supplementary Materials.

## Supplementary Materials

The following Supplementary Materials can be downloaded at: https://www.mdpi.com/article/10.3390/biom13081168/s1, Figure S1: Section of the MS spectrum of cucurbitacin B standard; Figure S2: Section of the MS spectrum of cucurbitacin E standard; Figure S3: Section of the MS spectrum of hemslecin A standard; Figure S4: MS/MS spectrum of cucurbitacin B standard [M + FA − H]^−^ adduct (40 eV collision energy); Figure S5: MS/MS spectrum of cucurbitacin E standard [M + FA − H]^−^ adduct (40 eV collision energy); Figure S6: Full TIC chromatogram of the extract; Figure S7. MS/MS spectrum of putatively identified cucurbitacin G/H [M + FA − H]^−^ adduct (40 eV collision energy); Figure S7. MS/MS spectrum of putatively identified cucurbitacin G/H [M + FA − H]^−^ adduct (40 eV collision energy); Figure S8. MS/MS spectrum of putatively identified cucurbitacin G/H [M + FA − H]^−^ adduct (40 eV collision energy); Figure S9. MS/MS spectrum of putatively identified cucurbitacin G/H [M + FA − H]^−^ adduct (40 eV collision energy); Figure S10. MS/MS spectrum of putatively identified cucurbitacin G/H [M + FA − H]^−^ adduct (40 eV collision energy); Figure S11. MS/MS spectrum of putatively identified cucurbitacin G/H [M + FA − H]^−^ adduct (40 eV collision energy); Figure S12. MS/MS spectrum of putatively identified cucurbitacin G/H [M + FA − H]^−^ adduct (40 eV collision energy); Figure S13. MS/MS spectrum of putatively identified cucurbitacin G/H [M + FA − H]^−^ adduct (40 eV collision energy); Figure S14. MS/MS spectrum of putatively identified cucurbitacin G/H [M + FA − H]^−^ adduct (40 eV collision energy); Figure S15. MS/MS spectrum of putatively identified cucurbitacin G/H [M + FA − H]^−^ adduct (40 eV collision energy); Figure S16. MS/MS spectrum of putatively identified cucurbitacin G/H [M + FA − H]^−^ adduct (40 eV collision energy); Figure S17. MS/MS spectrum of putatively identified cucurbitacin G/H [M + FA − H]^−^ adduct (40 eV collision energy); Figure S18. MS/MS spectrum of putatively identified cucurbitacin G/H [M + FA − H]^−^ adduct (40 eV collision energy); Table S1. Compound list with putatively identified (23,24-dihydro)cucurbitacins in grey.
