# Supplementary material for: Mass Spectrometric Analysis of Cucurbitacins and Dihydrocucurbitacins from the Tuber of Citrullus naudinianus
Source: Biomolecules. 2023 Jul 26;13(8):1168. doi: 10.3390/biom13081168 (PMC10452186; doi:10.3390/biom13081168)
Supplement: Supplementary file 1 [file biomolecules-13-01168-s001.zip › biomolecules-2425775-supplementary.pdf]

Article

Mass Spectrometric Analysis of Cucurbitacins and Dihydrocucurbitacins from the Tuber of *Citrullus naudinianus*

Moritz Benka [1,2,3], Kristof Görlitz [1], Michael C. Schöttgen [1,4], Simon Lagies [1,5], Daniel A. Mohl [1,2,3], Michel Kather [1], Iwanette Du Preez-Bruwer [6], Davis Mumbengegwi [6], Robin Teufel [7], Stefanie Kowarschik [4], Roman Huber [4], Dietmar A. Plattner [2,*] and Bernd Kammerer [1,2,8,*]

1 Core Competence Metabolomics, Hilde-Mangold-Haus, University of Freiburg, 79104 Freiburg, Germany; moritz.benka@ocbc.uni-freiburg.de (M.B.); kgoerlitz.1207@gmail.com (K.G.); michael.camillo.schoettgen@uniklinik-freiburg.de (M.C.S.); simon.lagies@oc.uni-freiburg.de (S.L.); daniel.mohl@ocbc.uni-freiburg.de (D.A.M.); kather-michel@web.de (M.K.)

2 Institute of Organic Chemistry, University of Freiburg, 79104 Freiburg, Germany

3 Hermann Staudinger Graduate School, University of Freiburg, 79104 Freiburg, Germany

4 Center for Complementary Medicine, Department of Internal Medicine II, University Hospital, Faculty of Medicine, University of Freiburg, 79106 Freiburg, Germany; stefanie.kowarschik@uniklinik-freiburg.de (S.K.); roman.huber@uniklinik-freiburg.de (R.H.)

5 Institute of Medical Microbiology and Hygiene, Faculty of Medicine, Medical Center—University of Freiburg, 79104 Freiburg, Germany

6 Centre for Research Services, University of Namibia, Private Bag 13301, Mandume, Ndemufayo Avenue, Pioneers Park, Windhoek, Namibia; ibruwer@unam.na (I.D.P.-B.); dmumbengegwi@unam.na (D.M.)

7 Department of Pharmaceutical Sciences, University of Basel, 4056 Basel, Switzerland; robin.teufel@unibas.ch

8 BIOSS Centre for Biological Signalling Studies, University of Freiburg, 79104 Freiburg, Germany

* Correspondence: dietmar.plattner@oc.uni-freiburg.de (D.A.P.); bernd.kammerer@oc.uni-freiburg.de (B.K.)

Supplementary Materials

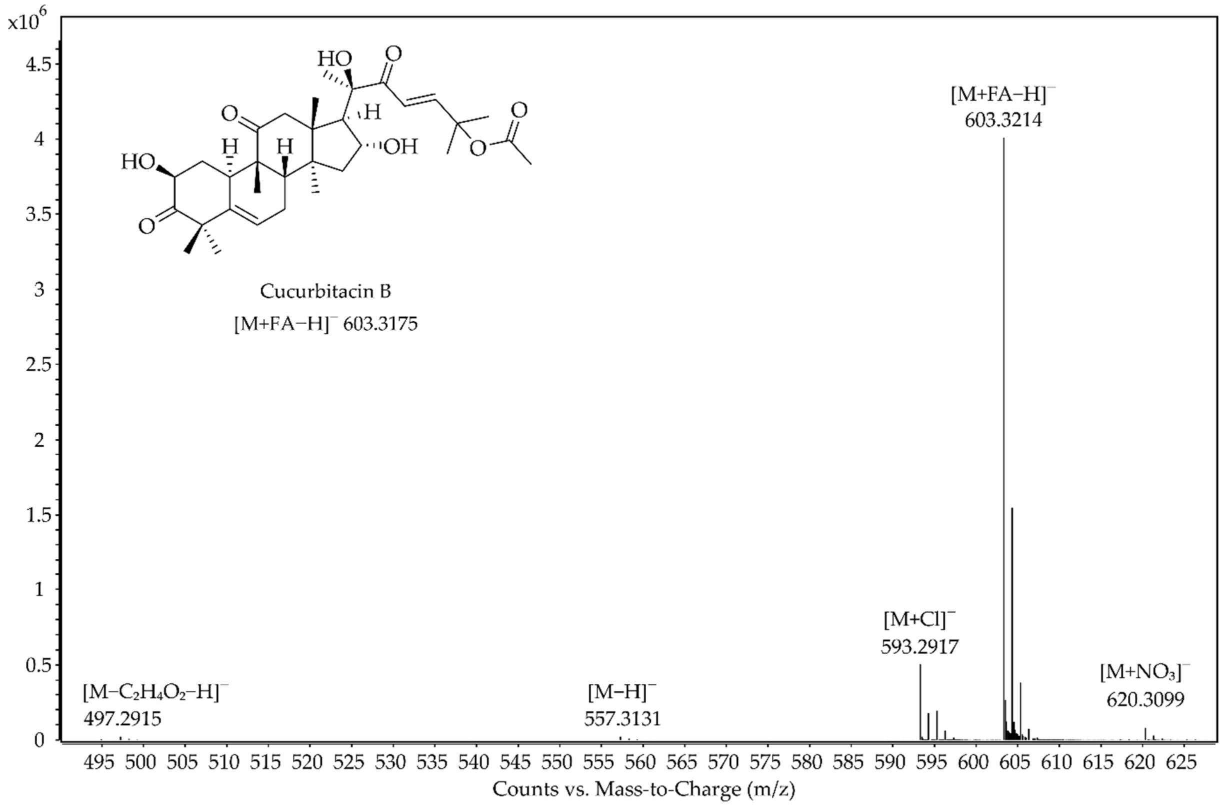

Figure S1. Section of the MS spectrum of cucurbitacin B standard.

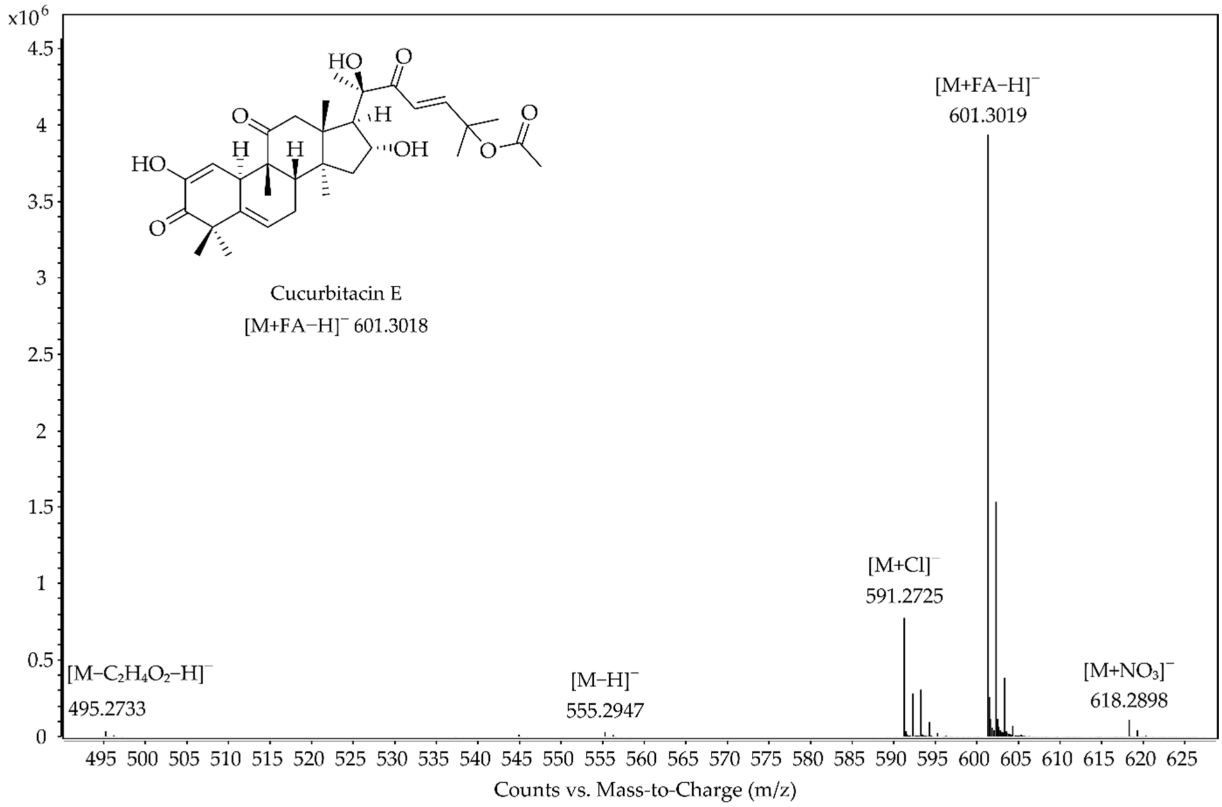

Figure S2. Section of the MS spectrum of cucurbitacin E standard.

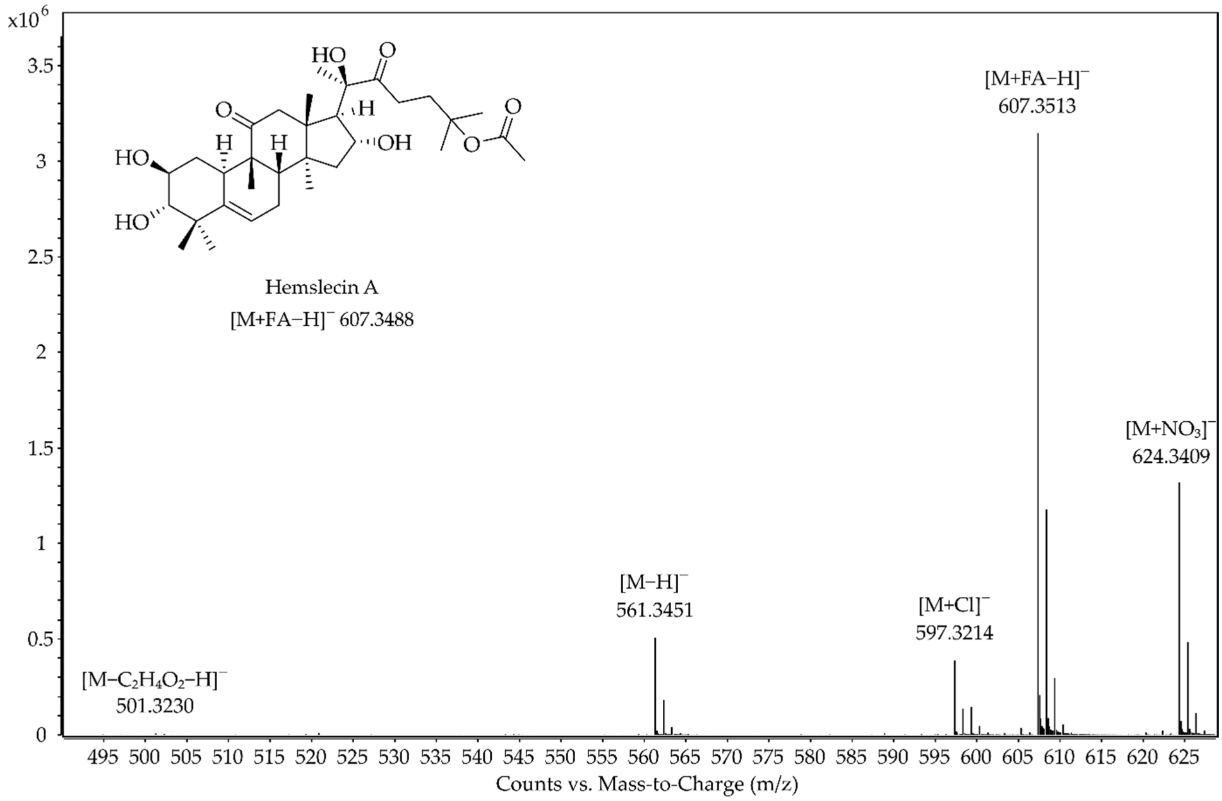

Figure S3. Section of the MS spectrum of hemslecin A standard.

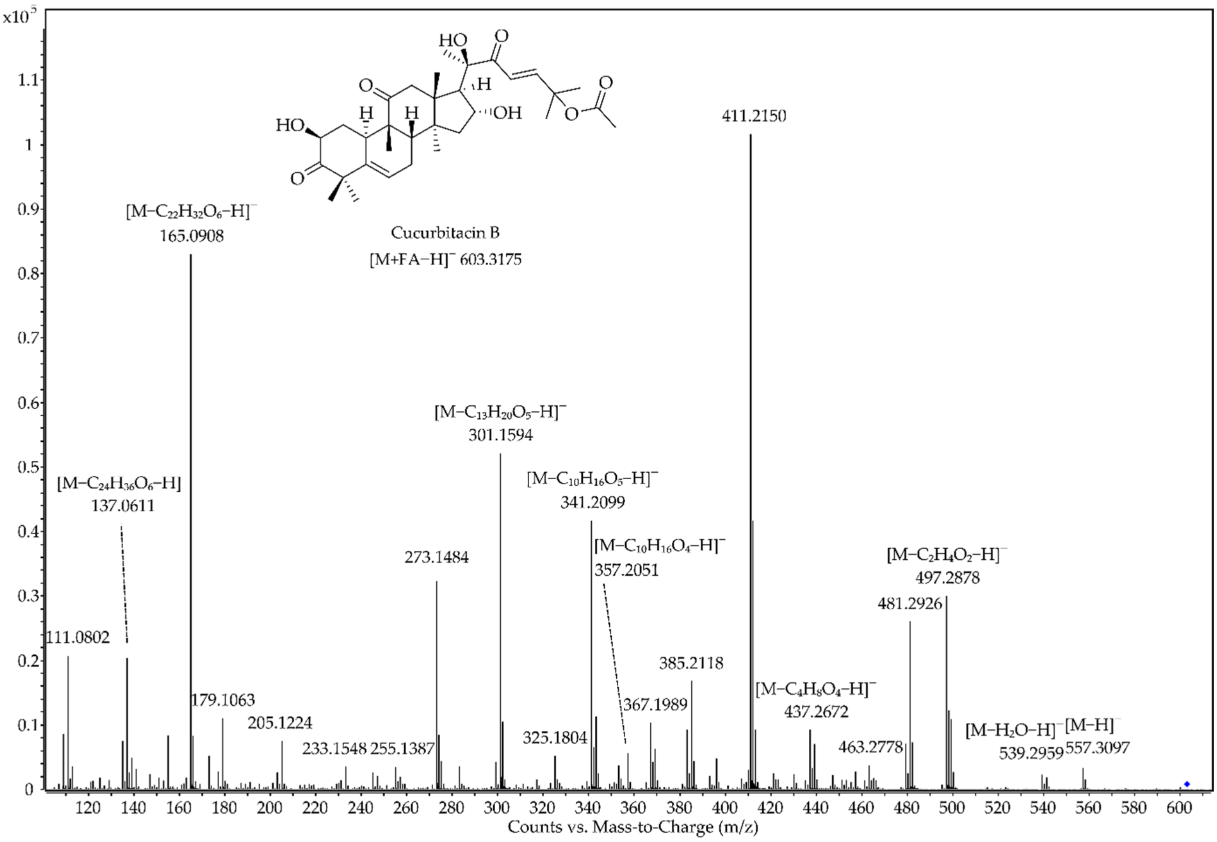

Figure S4. MS/MS spectrum of cucurbitacin B standard [M + FA − H]⁻ adduct (40 eV collision energy).

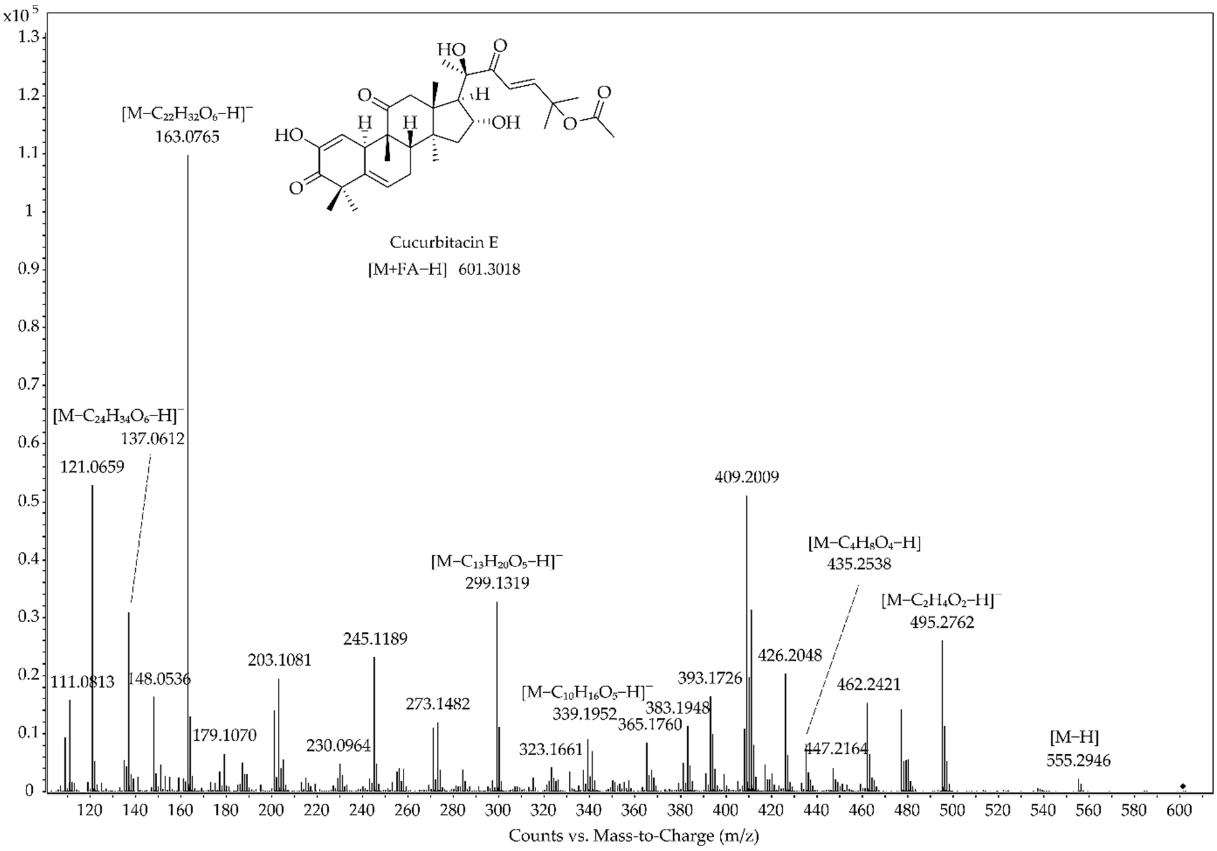

Figure S5. MS/MS spectrum of cucurbitacin E standard [M + FA − H]⁻ adduct (40 eV collision energy).

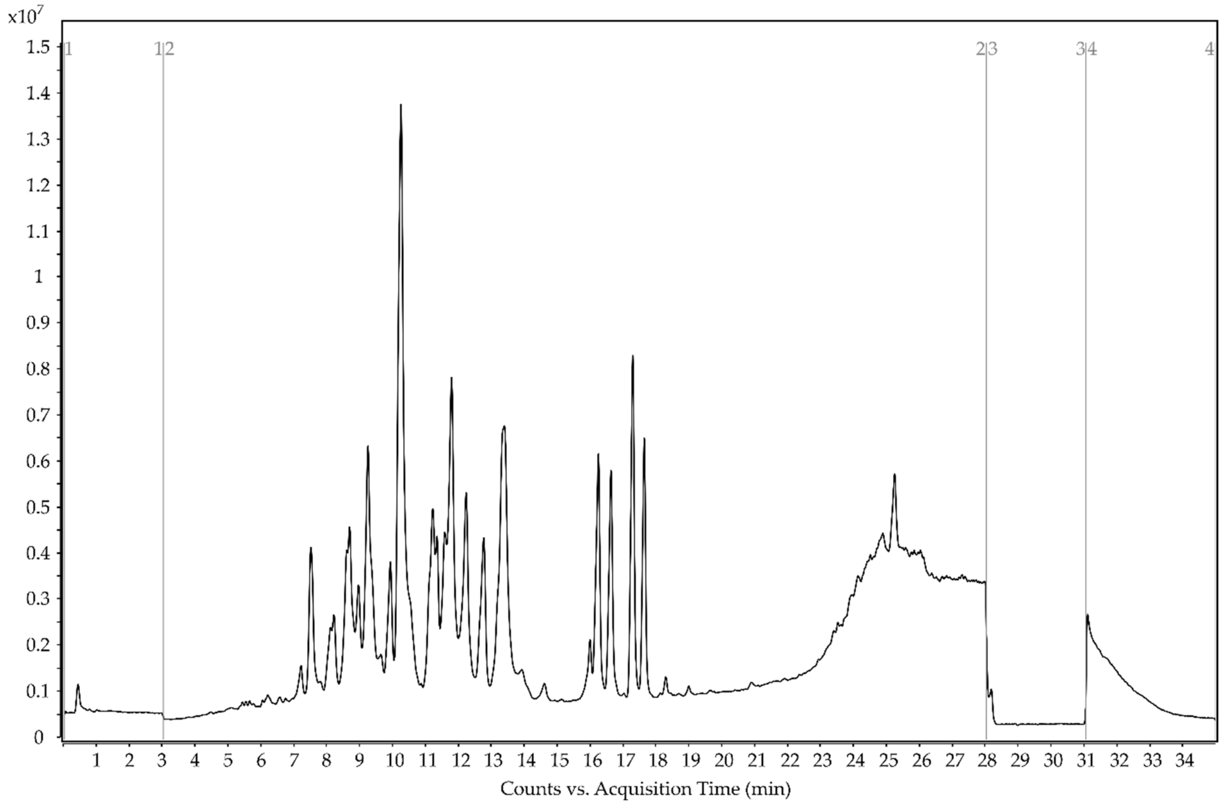

Figure S6. Full TIC chromatogram of the extract.

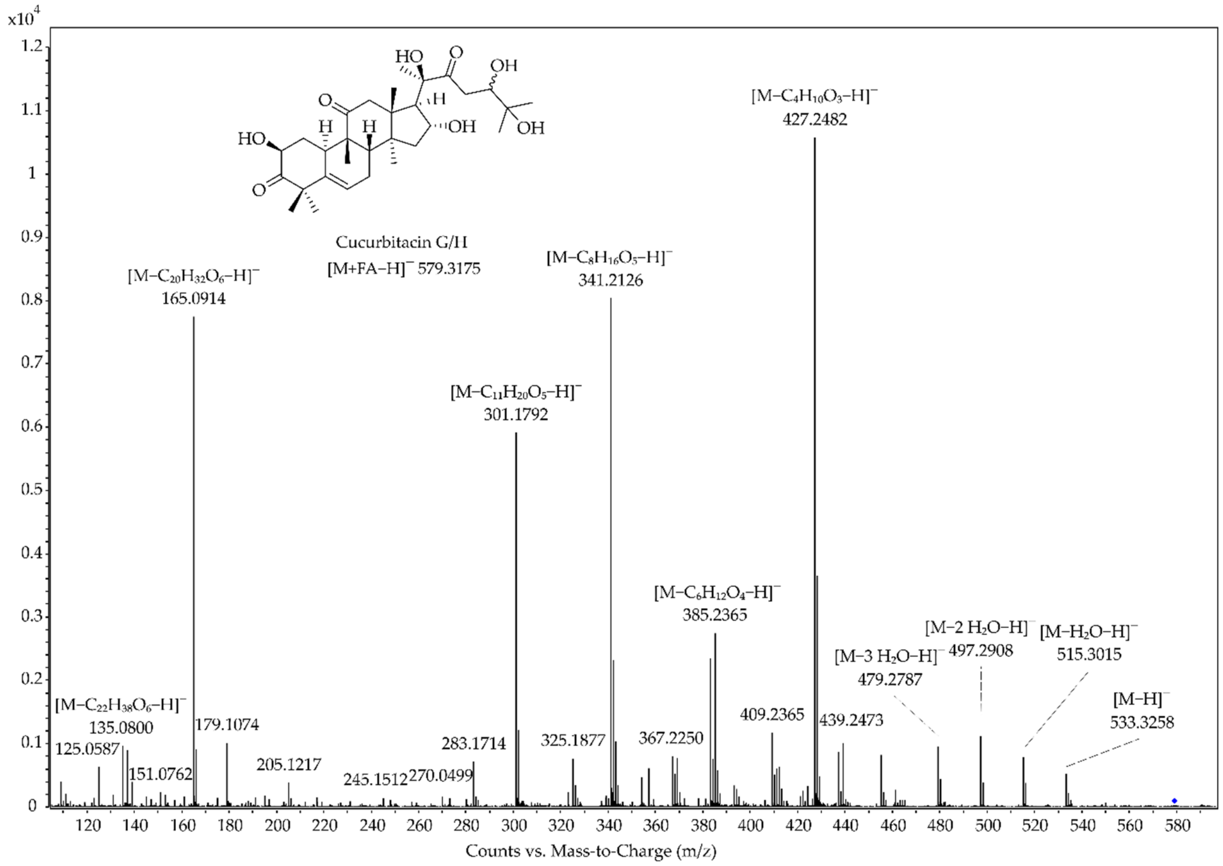

Figure S7. MS/MS spectrum of putatively identified cucurbitacin G/H [M + FA − H]⁻ adduct (40 eV collision energy).

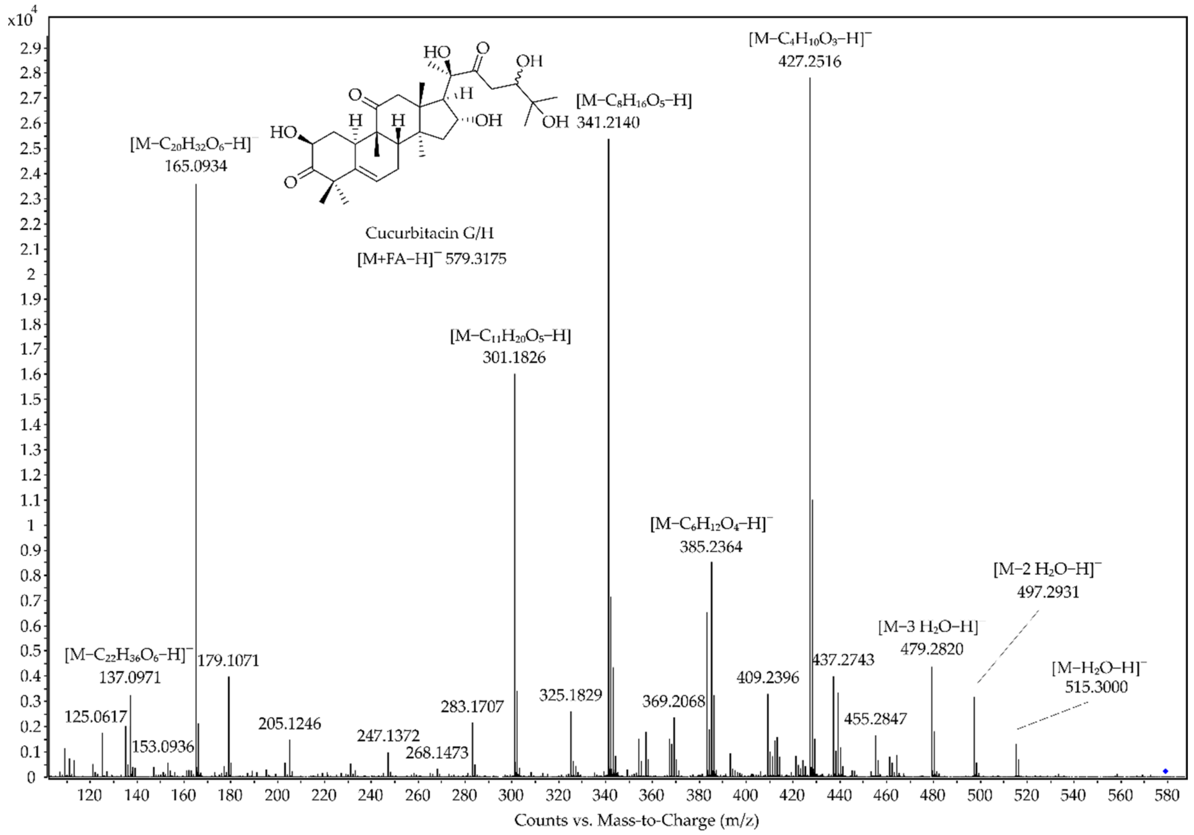

Figure S8. MS/MS spectrum of putatively identified cucurbitacin G/H [M + FA − H]⁻ adduct (40 eV collision energy).

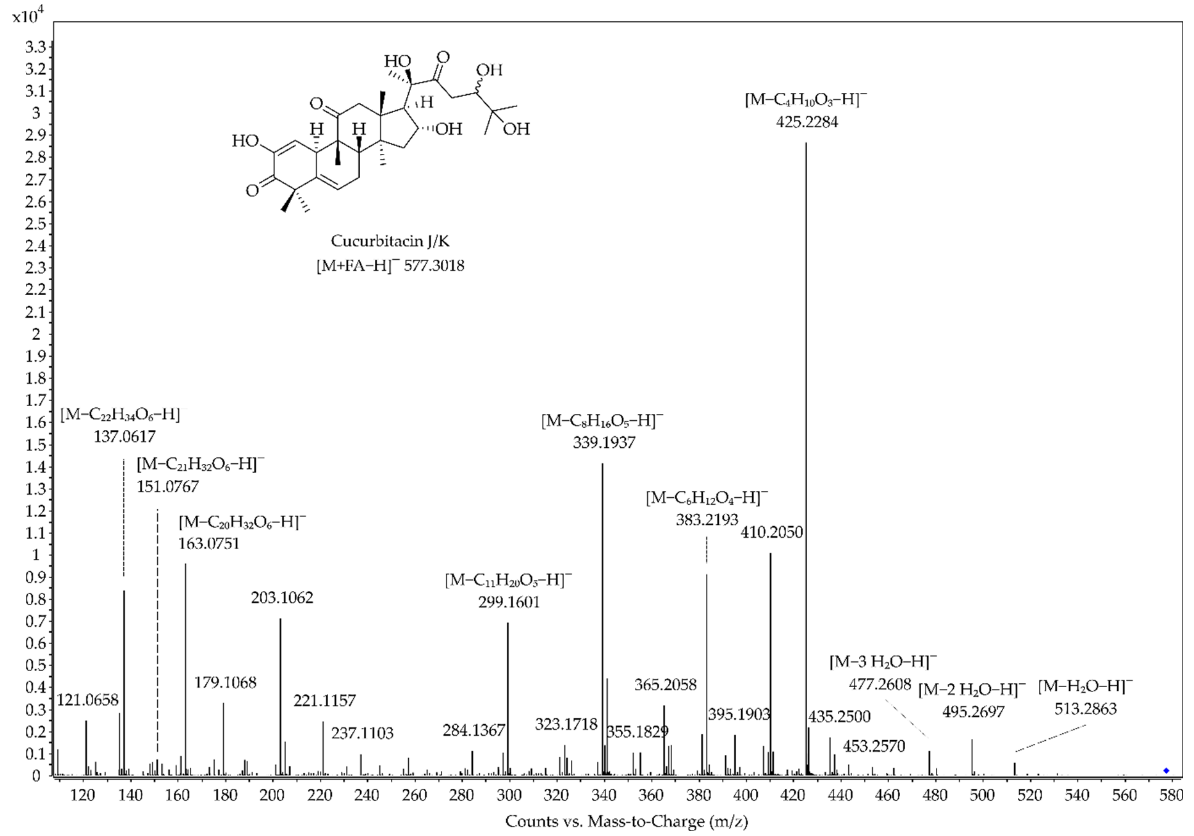

Figure S9. MS/MS spectrum of putatively identified cucurbitacin J/K [M + FA − H]⁻ adduct (40 eV collision energy).

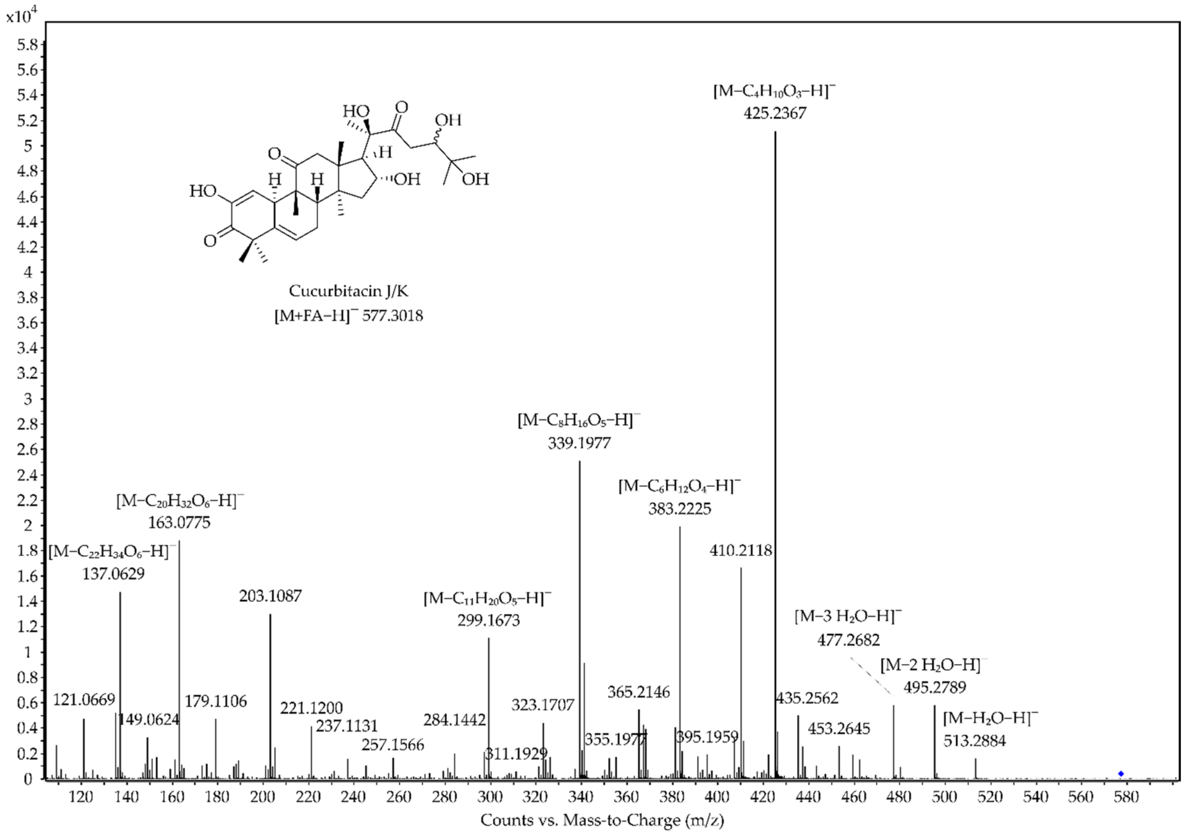

Figure S10. MS/MS spectrum of putatively identified cucurbitacin J/K [M + FA − H]⁻ adduct (40 eV collision energy).

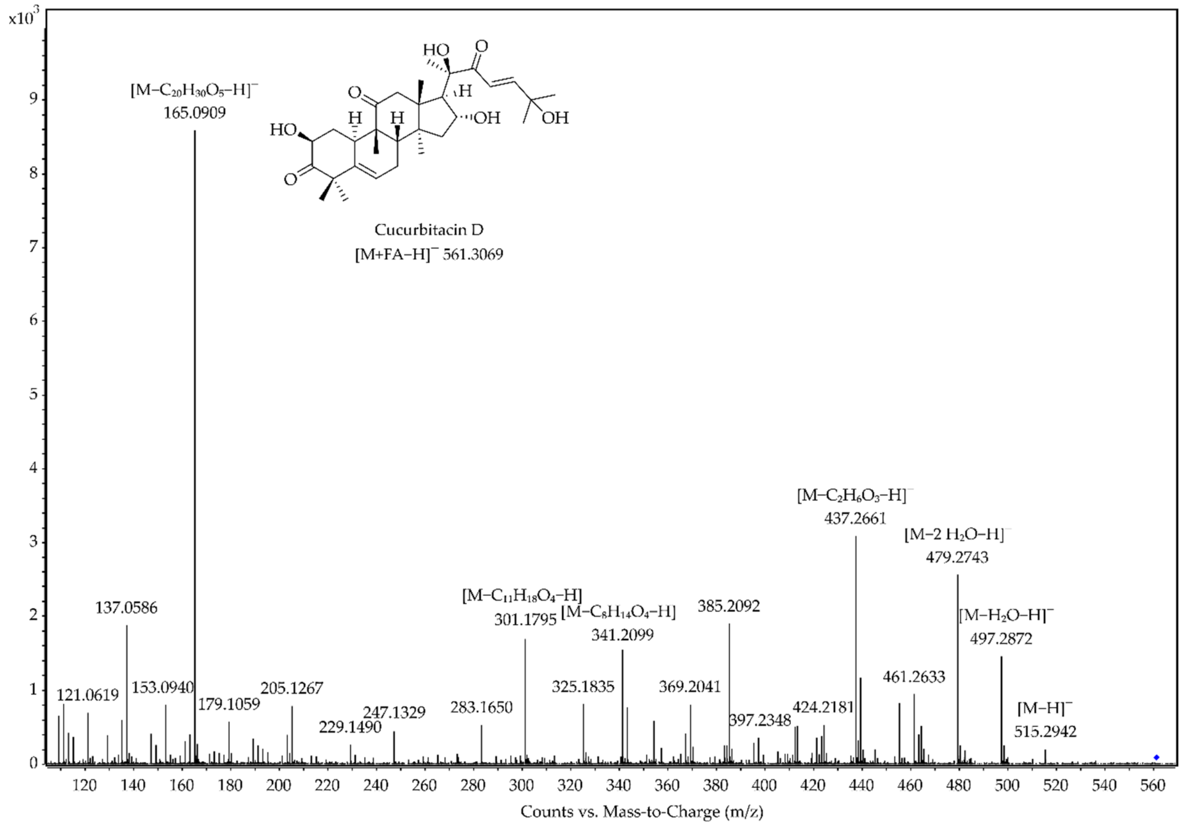

Figure S11. MS/MS spectrum of putatively identified cucurbitacin D [M + FA − H]⁻ adduct (40 eV collision energy).

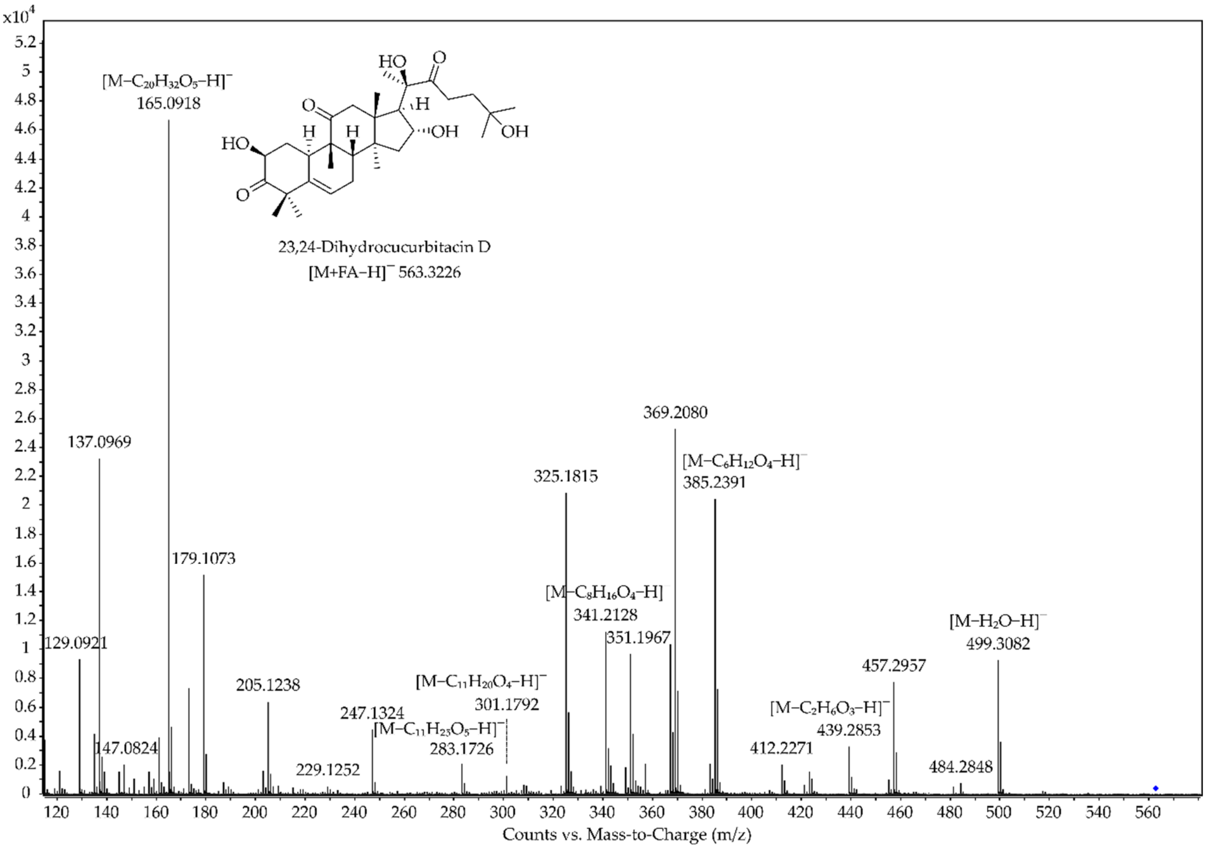

Figure S12. MS/MS spectrum of putatively identified 23,24-dihydrocucurbitacin D [M + FA − H]⁻ adduct (40 eV collision energy).

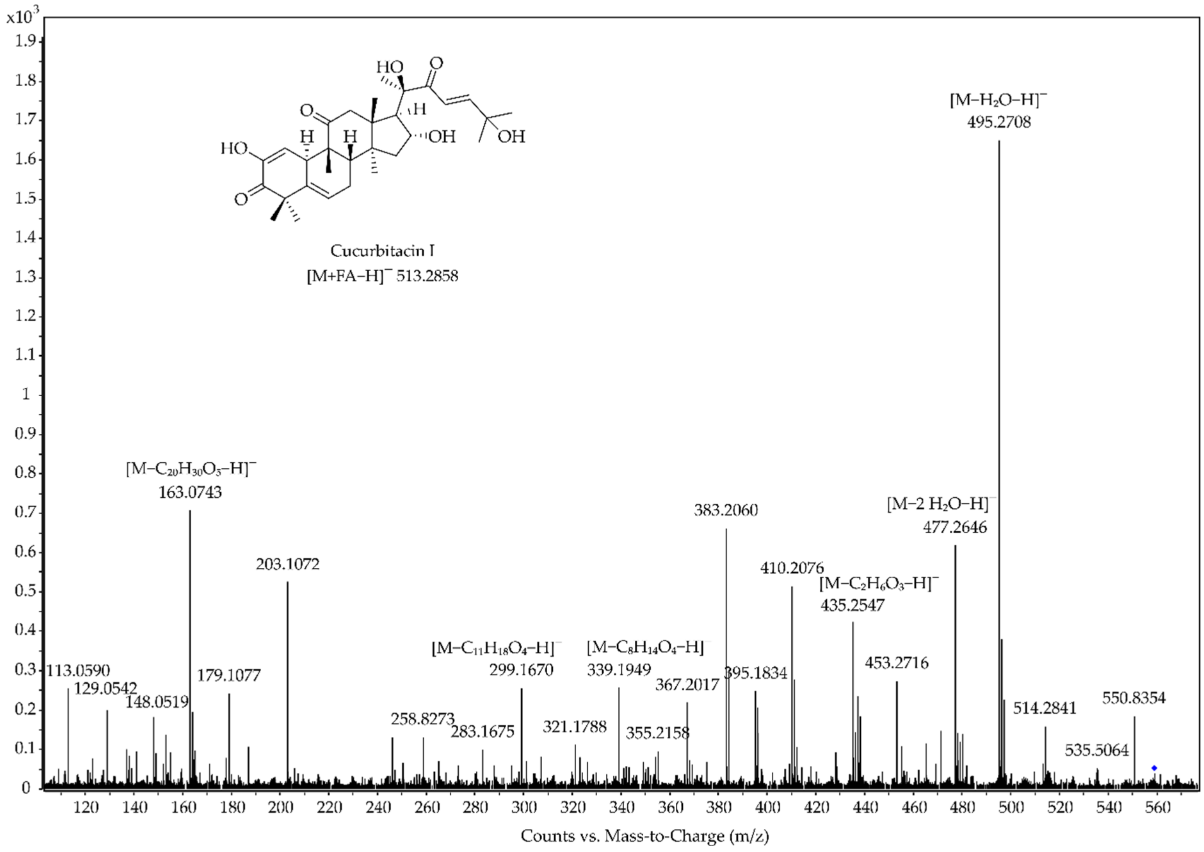

Figure S13. MS/MS spectrum of putatively identified cucurbitacin I [M + FA − H]⁻ adduct (40 eV collision energy).

.

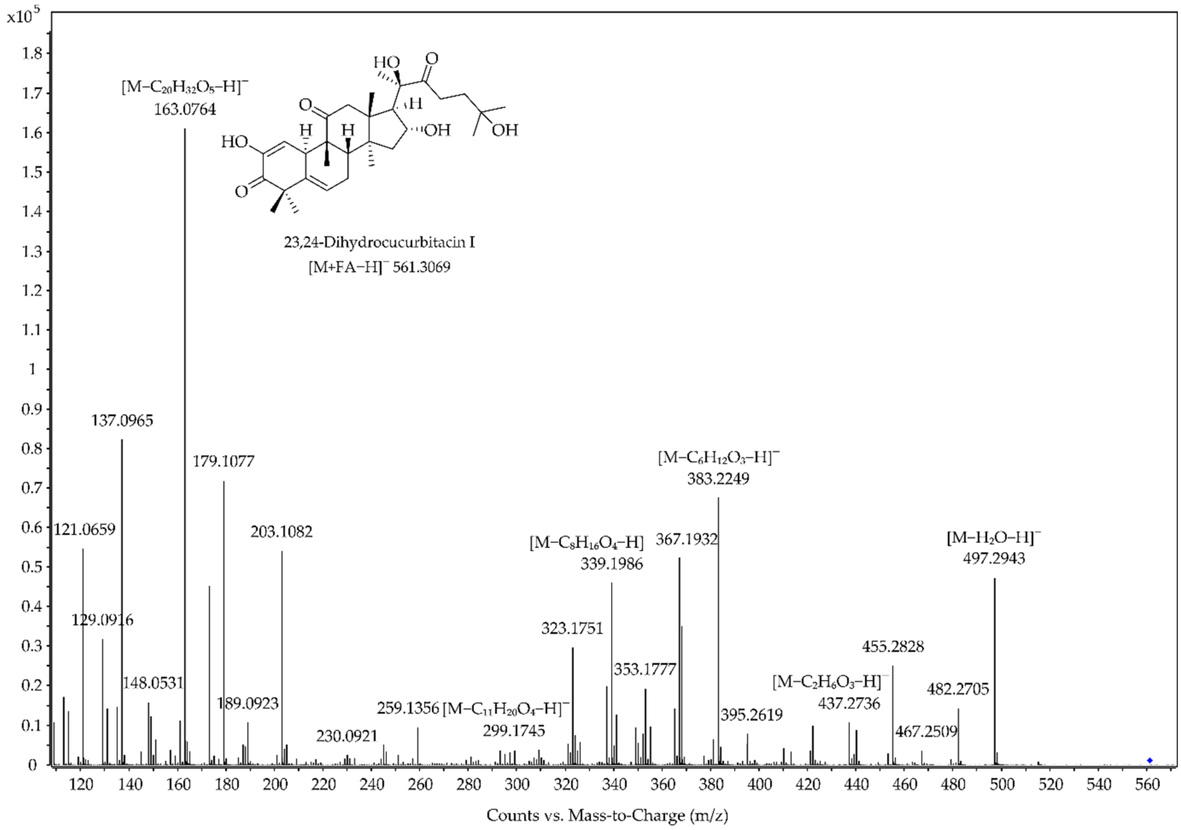

Figure S14. MS/MS spectrum of putatively identified 23,24-cucurbitacin I [M + FA − H]⁻ adduct (40 eV collision energy).

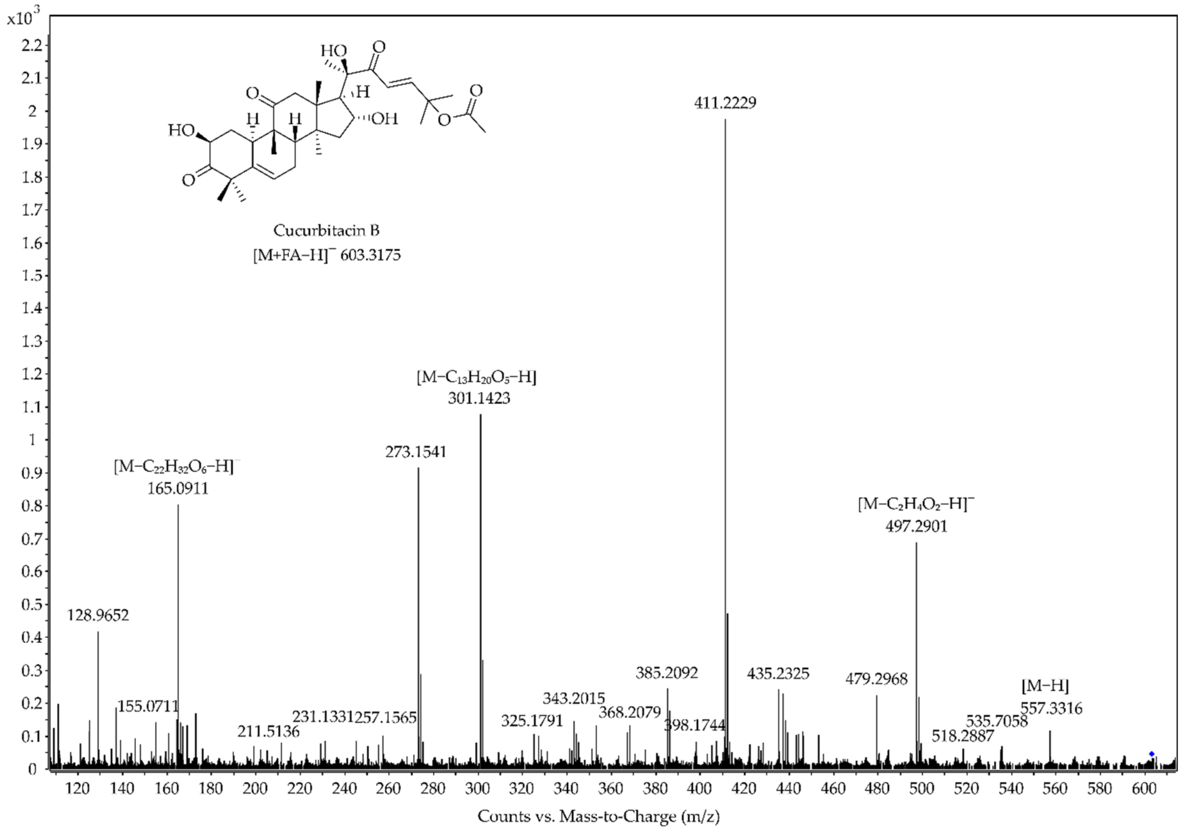

Figure S15. MS/MS spectrum of putatively identified cucurbitacin B [M + FA − H]⁻ adduct (40 eV collision energy).

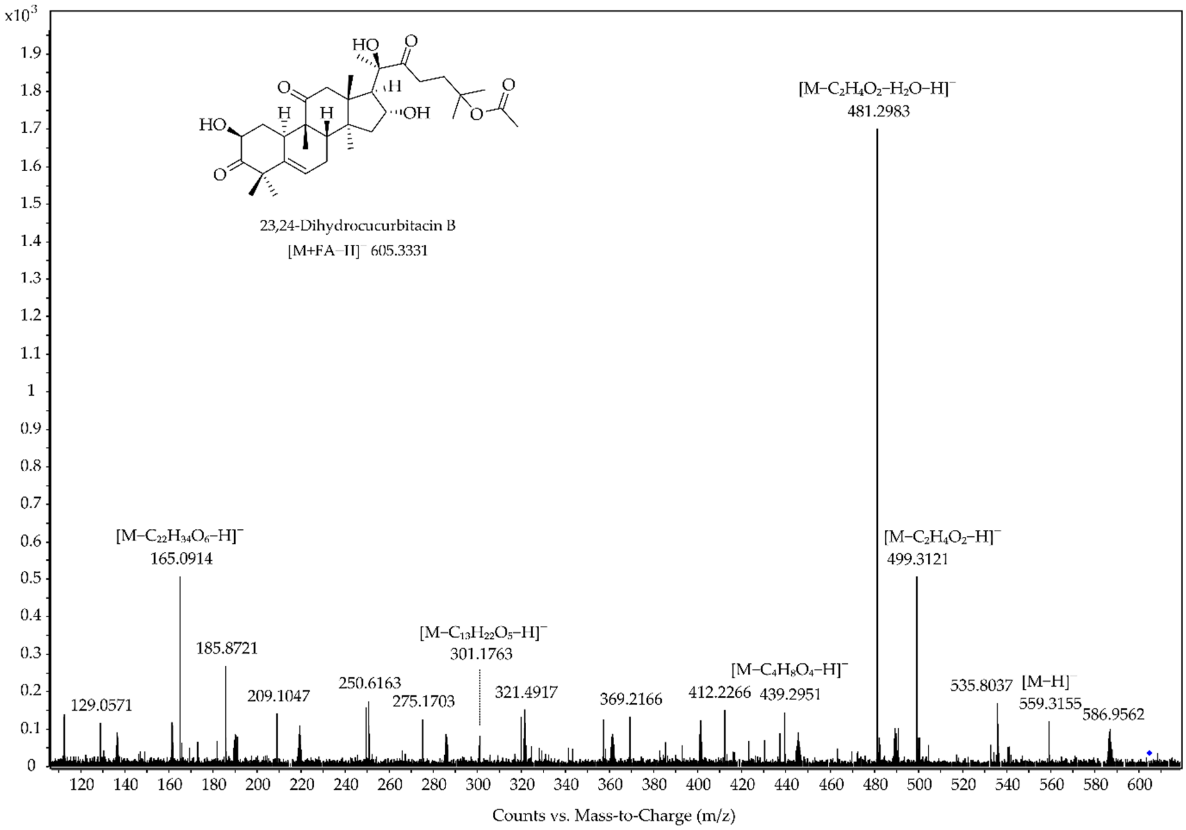

Figure S16. MS/MS spectrum of putatively identified 23,24-cucurbitacin B [M + FA − H]⁻ adduct (40 eV collision energy).

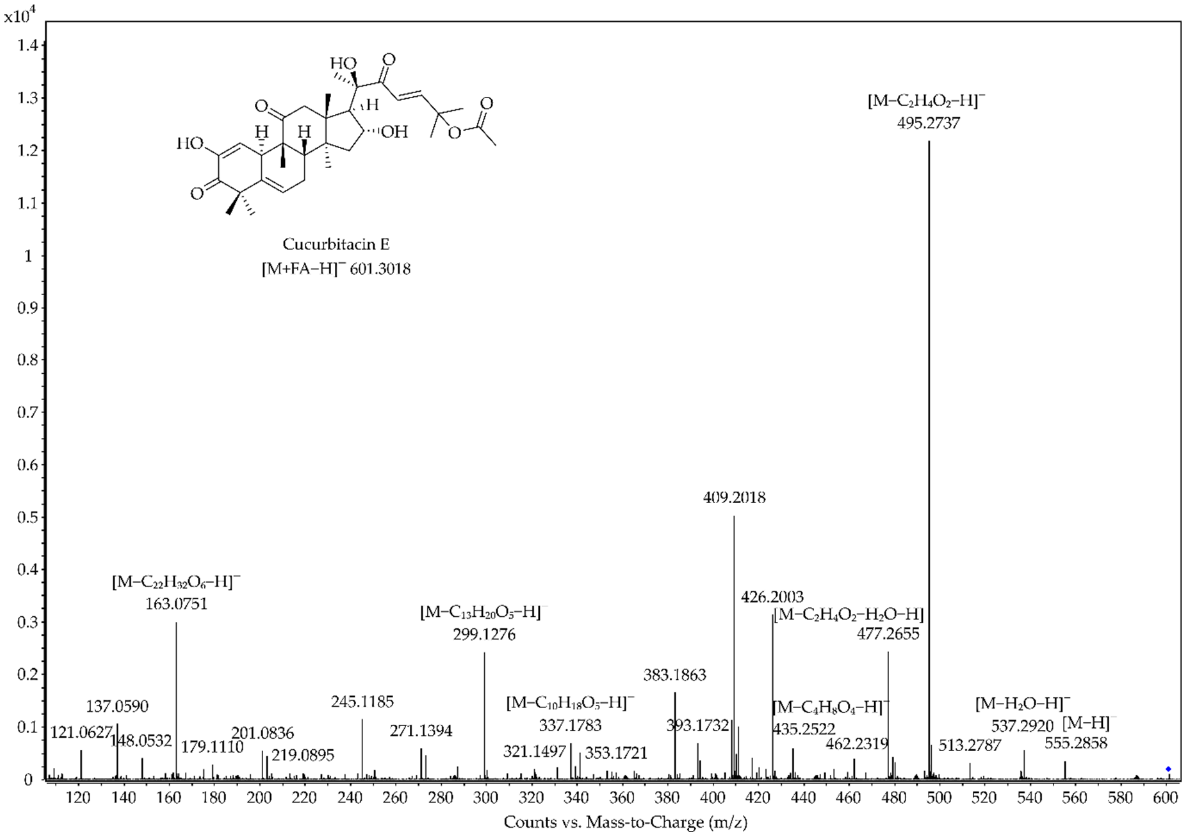

Figure S17. MS/MS spectrum of putatively identified cucurbitacin E [M + FA − H]⁻ adduct (40 eV collision energy).

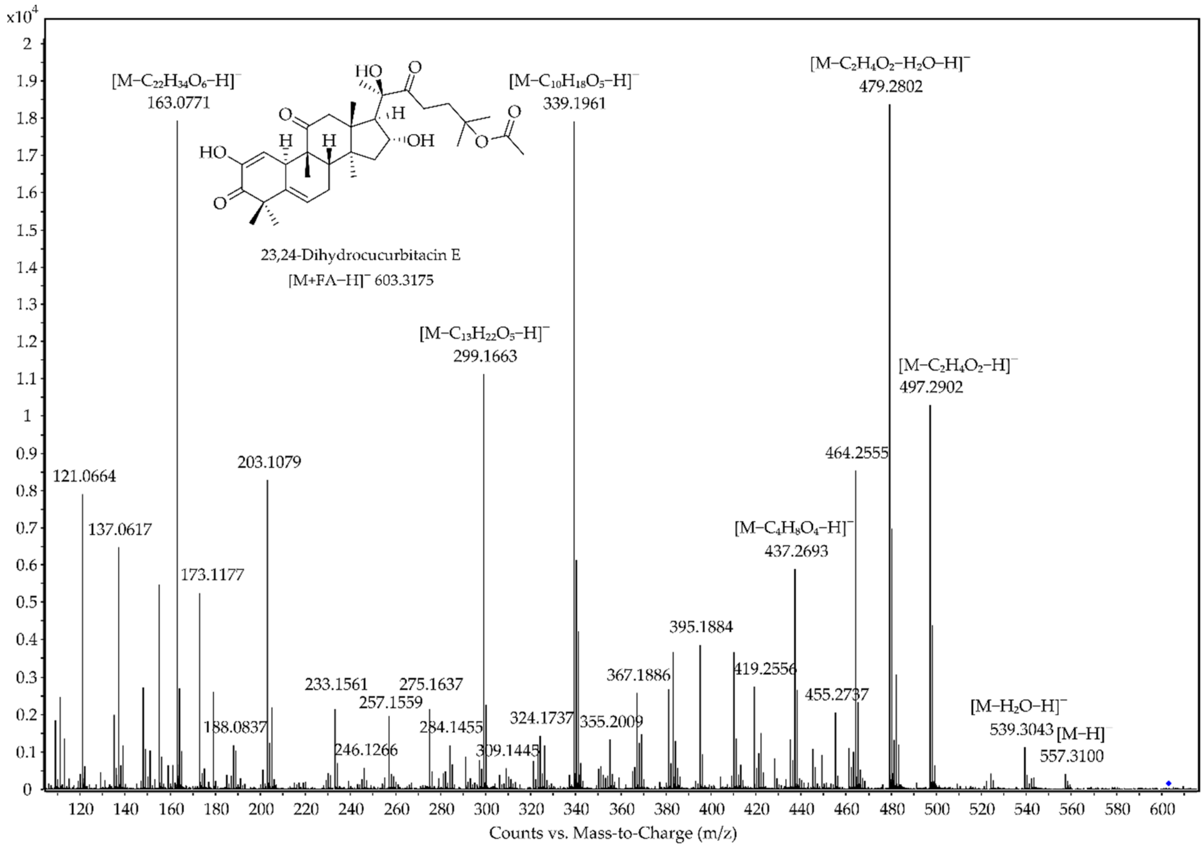

Figure S18. MS/MS spectrum of putatively identified 23,24-cucurbitacin E [M + FA − H]⁻ adduct (40 eV collision energy).

Table S1. Compound list with putatively identified (23,24-dihydro)cucurbitacins in grey.

Compound No.	RT/min	Formula	*m*/*z*	Most Abundant (Adduct) Ion	Exact Mass	Error /ppm	Height	Volume
1	7.16	$C_{42}H_{70}O_{16}$	865.4337	[M + Cl]⁻	865.4358	−2.43	25814	127483
2	7.22	$C_{36}H_{56}O_{12}$	725.3755	[M + FA − H]⁻	725.3754	0.14	64746	447110
3	7.22	$C_{30}H_{42}O_{10}$	597.2478	[M + Cl]⁻	597.2472	1.00	61796	797931
4	7.23	$C_{42}H_{66}O_{16}$	871.4335	[M + FA − H]⁻	871.4333	0.23	79491	651897
5	7.52	$C_{32}H_{46}O_{11}$	605.2972	[M − H]⁻	605.2967	0.83	29785	196739
6	7.53	$C_{36}H_{54}O_{13}$	739.3567	[M + FA − H]⁻	739.3546	2.84	729093	10040805
7	7.84	$C_{42}H_{64}O_{15}$	853.4240	[M + FA − H]⁻	853.4227	1.52	51821	334692
8	8.02	$C_{36}H_{54}O_{12}$	723.3611	[M + FA − H]⁻	723.3597	1.94	89401	656056
9	8.08	$C_{36}H_{56}O_{13}$	741.3706	[M + FA − H]⁻	741.3703	0.40	148668	1442574
10	8.12	$C_{42}H_{68}O_{17}$	889.4424	[M + FA − H]⁻	889.4439	−1.69	26595	158154
11	8.12	$C_{42}H_{62}O_{15}$	851.4084	[M + FA − H]⁻	851.4071	1.53	214222	1734968
12	8.22	$C_{36}H_{56}O_{13}$	741.3704	[M + FA − H]⁻	741.3703	0.13	143709	1212229
13	8.22	$C_{42}H_{64}O_{15}$	853.4233	[M + FA − H]⁻	853.4227	0.70	329859	4006447
14	8.27	$C_{42}H_{68}O_{15}$	857.4526	[M + FA − H]⁻	857.4540	−1.63	31885	262591
15	8.46	$C_{42}H_{70}O_{14}$	843.4742	[M + FA − H]⁻	843.4748	−0.71	64074	500350
16	8.51	$C_{42}H_{66}O_{15}$	855.4370	[M + FA − H]⁻	855.4384	−1.64	23663	118273
17	8.52	$C_{42}H_{68}O_{16}$	873.4497	[M + FA − H]⁻	873.4489	0.92	100870	946237
18	8.58	$C_{36}H_{52}O_{12}$	721.3465	[M + FA − H]⁻	721.3441	3.33	591250	5025215
19	8.61	$C_{36}H_{56}O_{12}$	725.3735	[M + FA − H]⁻	725.3754	−2.62	103560	713091
20	8.62	$C_{42}H_{70}O_{15}$	859.4708	[M + FA − H]⁻	859.4697	1.28	166140	2727058
21	8.70	$C_{36}H_{54}O_{12}$	723.3613	[M + FA − H]⁻	723.3597	2.21	1090731	14113380
22	8.80	$C_{48}H_{78}O_{20}$	973.5011	[M − H]⁻	973.5014	−0.31	129919	3130065
23	8.91	$C_{36}H_{52}O_{11}$	705.3504	[M + FA − H]⁻	705.3492	1.70	35347	754394
24	8.93	$C_{38}H_{56}O_{14}$	781.3659	[M + FA − H]⁻	781.3652	0.90	34658	359561

25	8.95	$C_{36}H_{54}O_{11}$	707.3651	$[M + FA - H]^-$	707.3648	0.42	31779	211092
26	8.96	$C_{42}H_{62}O_{15}$	851.4093	$[M + FA - H]^-$	851.4071	2.58	496474	5335848
27	9.02	$C_{42}H_{68}O_{16}$	863.4201	$[M + Cl]^-$	863.4201	0.00	133557	3382514
28	9.14	$C_{42}H_{68}O_{14}$	841.4585	$[M + FA - H]^-$	841.4591	−0.71	167364	1764773
29	9.19	$C_{42}H_{70}O_{15}$	859.4691	$[M + FA - H]^-$	859.4697	−0.70	153536	1381259
30	9.22	$C_{39}H_{60}O_{14}$	797.3974	$[M + FA - H]^-$	797.3965	1.13	32067	207755
31	9.25	$C_{42}H_{68}O_{16}$	873.4525	$[M + FA - H]^-$	873.4489	4.12	1530795	16659874
32	9.31	$C_{42}H_{70}O_{15}$	859.4704	$[M + FA - H]^-$	859.4697	0.81	231748	2354448
33	9.38	$C_{36}H_{56}O_{12}$	715.3470	$[M + Cl]^-$	715.3466	0.56	299862	3884532
34	9.39	$C_{42}H_{68}O_{15}$	857.4548	$[M + FA - H]^-$	857.4540	0.93	63816	533628
35	9.42	$C_{36}H_{54}O_{12}$	723.3602	$[M + FA - H]^-$	723.3597	0.69	154382	1464379
36	9.57	$C_{36}H_{54}O_{11}$	707.3666	$[M + FA - H]^-$	707.3648	2.54	27561	179466
37	9.64	$C_{42}H_{70}O_{16}$	875.4661	$[M + FA - H]^-$	875.4646	1.71	63026	545103
38	9.66	$C_{48}H_{78}O_{19}$	993.4836	$[M + Cl]^-$	993.4831	0.50	44040	1024727
39	9.85	$C_{42}H_{66}O_{15}$	855.4390	$[M + FA - H]^-$	855.4384	0.70	55691	611199
40	9.93	$C_{30}H_{46}O_8$	569.2898	$[M + Cl]^-$	569.2887	1.93	489643	16413932
41	9.96	$C_{42}H_{68}O_{15}$	857.4533	$[M + FA - H]^-$	857.4540	−0.82	55923	768545
42	10.01	$C_{45}H_{70}O_{19}$	913.4431	$[M - H]^-$	913.4439	−0.88	26527	172093
43	10.19	$C_{30}H_{46}O_8$	579.3179	$[M + FA - H]^-$	579.3175	0.69	253559	3531450
44	10.24	$C_{42}H_{70}O_{14}$	843.4739	$[M + FA - H]^-$	843.4748	−1.07	36557	404190
45	10.25	$C_{42}H_{68}O_{15}$	857.4558	$[M + FA - H]^-$	857.4540	2.10	4121930	68503056
46	10.47	$C_{42}H_{66}O_{14}$	839.4425	$[M + FA - H]^-$	839.4435	−1.19	25326	154928
47	10.54	$C_{42}H_{70}O_{15}$	859.4682	$[M + FA - H]^-$	859.4697	−1.75	183392	2144768
48	10.54	$C_{38}H_{56}O_{13}$	765.3708	$[M + FA - H]^-$	765.3703	0.65	49177	428542
49	10.55	$C_{42}H_{70}O_{15}$	849.4374	$[M + Cl]^-$	849.4409	−4.12	90853	1252815
50	10.62	$C_{48}H_{78}O_{18}$	941.5112	$[M - H]^-$	941.5115	−0.32	27394	304921
51	10.73	$C_{30}H_{46}O_7$	563.3224	$[M + FA - H]^-$	563.3226	−0.36	57812	546138
52	10.86	$C_{42}H_{62}O_{15}$	851.4085	$[M + FA - H]^-$	851.4071	1.64	39035	337817
53	11.09	$C_{45}H_{70}O_{18}$	897.4506	$[M - H]^-$	897.4489	1.89	512795	6253428
54	11.13	$C_{38}H_{58}O_{13}$	767.3869	$[M + FA - H]^-$	767.3859	1.30	33383	285582
55	11.14	$C_{36}H_{58}O_{11}$	711.3970	$[M + FA - H]^-$	711.3961	1.27	40627	340181
56	11.21	$C_{30}H_{44}O_8$	567.2749	$[M + Cl]^-$	567.2730	3.35	613830	18781654
57	11.34	$C_{42}H_{68}O_{14}$	841.4632	$[M + FA - H]^-$	841.4591	4.87	971265	12896669
58	11.39	$C_{45}H_{70}O_{18}$	897.4494	$[M - H]^-$	897.4489	0.56	42022	664776
59	11.57	$C_{30}H_{44}O_8$	567.2746	$[M + Cl]^-$	567.2730	2.82	673878	20494436
60	11.62	$C_{42}H_{60}O_{15}$	849.3939	$[M + FA - H]^-$	849.3914	2.94	46039	595385
61	11.77	$C_{30}H_{44}O_7$	561.3090	$[M + FA - H]^-$	561.3069	3.74	600292	7325956
62	11.80	$C_{30}H_{46}O_7$	563.3243	$[M + FA - H]^-$	563.3226	3.02	1096570	19759394
63	11.87	$C_{42}H_{66}O_{15}$	855.4393	$[M + FA - H]^-$	855.4384	1.05	30237	220472
64	12.04	$C_{30}H_{44}O_7$	561.3071	$[M + FA - H]^-$	561.3069	0.36	120461	1279097
65	12.05	$C_{44}H_{70}O_{16}$	899.4634	$[M + FA - H]^-$	899.4646	−1.33	22202	131310
66	12.19	$C_{45}H_{70}O_{17}$	881.4530	$[M - H]^-$	881.4540	−1.13	62632	669942
67	12.23	$C_{38}H_{54}O_{13}$	763.3558	$[M + FA - H]^-$	763.3546	1.57	1004586	29706660
68	12.25	$C_{42}H_{68}O_{13}$	825.4639	$[M + FA - H]^-$	825.4642	−0.36	195659	2243948
69	12.26	$C_{30}H_{46}O_6$	547.3277	$[M + FA - H]^-$	547.3276	0.18	41488	410160
70	12.39	$C_{38}H_{58}O_{13}$	767.3851	$[M + FA - H]^-$	767.3859	−1.04	32317	279112
71	12.60	$C_{42}H_{68}O_{14}$	841.4594	$[M + FA - H]^-$	841.4591	0.36	24190	160289
72	12.62	$C_{44}H_{70}O_{16}$	899.4651	$[M + FA - H]^-$	899.4646	0.56	49782	480523
73	12.76	$C_{38}H_{56}O_{13}$	765.3719	$[M + FA - H]^-$	765.3703	2.09	942462	14161664
74	13.16	$C_{38}H_{58}O_{13}$	767.3877	$[M + FA - H]^-$	767.3859	2.35	291049	3935643
75	13.31	$C_{30}H_{42}O_7$	559.2930	$[M + FA - H]^-$	559.2913	3.04	755152	9538335
76	13.41	$C_{30}H_{44}O_7$	551.2783	$[M + Cl]^-$	551.2781	0.36	1110845	48287100
77	13.56	$C_{36}H_{52}O_{11}$	705.3485	$[M + FA - H]^-$	705.3492	−0.99	38623	370979
78	13.85	$C_{42}H_{68}O_{14}$	841.4584	$[M + FA - H]^-$	841.4591	−0.83	38057	826216
79	13.92	$C_{30}H_{44}O_6$	545.3119	$[M + FA - H]^-$	545.3120	−0.18	37071	354331
80	14.15	$C_{42}H_{62}O_{14}$	835.4128	$[M + FA - H]^-$	835.4122	0.72	32461	319921
81	14.60	$C_{42}H_{62}O_{14}$	835.4123	$[M + FA - H]^-$	835.4122	0.12	80803	1056110
82	16.00	$C_{40}H_{56}O_{14}$	805.3648	$[M + FA - H]^-$	805.3652	−0.50	310791	3560361
83	16.18	$C_{30}H_{48}O_6$	549.3435	$[M + FA - H]^-$	549.3433	0.36	53980	419955

84	16.24	$C_{32}H_{46}O_8$	603.3190	$[M + FA - H]^-$	603.3175	2.49	1442206	16473799
85	16.63	$C_{32}H_{48}O_8$	605.3345	$[M + FA - H]^-$	605.3331	2.31	1231692	30598622
86	16.68	$C_{32}H_{46}O_8$	603.3174	$[M + FA - H]^-$	603.3175	−0.17	64680	625667
87	16.78	$C_{32}H_{44}O_8$	601.3015	$[M + FA - H]^-$	601.3018	−0.50	46695	324567
88	17.25	$C_{30}H_{44}O_6$	545.3126	$[M + FA - H]^-$	545.3120	1.10	54047	398402
89	17.29	$C_{32}H_{44}O_8$	601.3034	$[M + FA - H]^-$	601.3018	2.66	2215801	20875588
90	17.64	$C_{32}H_{46}O_8$	603.3185	$[M + FA - H]^-$	603.3175	1.66	1372529	29244340
91	17.65	$C_{33}H_{46}O_{10}$	601.3016	$[M - H]^-$	601.3018	−0.33	69070	582316
92	18.30	$C_{30}H_{42}O_6$	543.2958	$[M + FA - H]^-$	543.2963	−0.92	87321	550549
93	19.00	$C_{34}H_{46}O_9$	643.3115	$[M + FA - H]^-$	643.3124	−1.40	62152	399196
94	24.78	$C_{35}H_{60}O_6$	621.4380	$[M + FA - H]^-$	621.4372	1.29	53075	378429
95	24.88	$C_{39}H_{54}O_5$	637.3672	$[M + Cl]^-$	637.3665	1.10	164119	2091066
